# Chromatin remodeler BAF maintains HBV cccDNA transcriptional competence and represents a therapeutic target

**DOI:** 10.1093/nar/gkag073

**Published:** 2026-02-10

**Authors:** Dan Huang, Yi Zheng, Enze Deng, Xinlei Ji, Yecheng Zhang, Hao Sun, Yingshan Chen, Yongxuan Yao, Yuan Zhou, Mingxia Zhang, Zhe Zhou, Yinghua Chen, Dan Su, Xiaoying Fan, Xinwen Chen, Rongjuan Pei

**Affiliations:** Key Laboratory of Virology and Biosafety, Wuhan Institute of Virology, Chinese Academy of Sciences, Wuhan 430071, China; Medical School, University of Chinese Academy of Sciences, Beijing 100049, China; Guangzhou National Laboratory, Guangzhou International Bio Island, Guangzhou 510005, China; Key Laboratory of Virology and Biosafety, Wuhan Institute of Virology, Chinese Academy of Sciences, Wuhan 430071, China; Medical School, University of Chinese Academy of Sciences, Beijing 100049, China; Guangzhou National Laboratory, Guangzhou International Bio Island, Guangzhou 510005, China; MOE Key Laboratory of Gene Function and Regulation, Guangdong Province Key Laboratory of Pharmaceutical Functional Genes, State Key Laboratory of Biocontrol, School of Life Sciences, Sun Yat-sen University, Guangzhou 510275, China; Key Laboratory of Virology and Biosafety, Wuhan Institute of Virology, Chinese Academy of Sciences, Wuhan 430071, China; Medical School, University of Chinese Academy of Sciences, Beijing 100049, China; Guangzhou National Laboratory, Guangzhou International Bio Island, Guangzhou 510005, China; Key Laboratory of Virology and Biosafety, Wuhan Institute of Virology, Chinese Academy of Sciences, Wuhan 430071, China; Institute of Pediatrics, Guangzhou Women and Children’s Medical Center, Guangzhou Medical University, Guangzhou 510623, China; Key Laboratory of Virology and Biosafety, Wuhan Institute of Virology, Chinese Academy of Sciences, Wuhan 430071, China; Medical School, University of Chinese Academy of Sciences, Beijing 100049, China; Key Laboratory of Virology and Biosafety, Wuhan Institute of Virology, Chinese Academy of Sciences, Wuhan 430071, China; Key Laboratory of Virology and Biosafety, Wuhan Institute of Virology, Chinese Academy of Sciences, Wuhan 430071, China; Guangzhou National Laboratory, Guangzhou International Bio Island, Guangzhou 510005, China; Key Laboratory of Virology and Biosafety, Wuhan Institute of Virology, Chinese Academy of Sciences, Wuhan 430071, China; Key Laboratory of Virology and Biosafety, Wuhan Institute of Virology, Chinese Academy of Sciences, Wuhan 430071, China; Organ Transplant, The First Affiliated Hospital, Sun Yat-sen University, Guangzhou 510080, China; Guangzhou National Laboratory, Guangzhou International Bio Island, Guangzhou 510005, China; Guangzhou National Laboratory, Guangzhou International Bio Island, Guangzhou 510005, China; GMU-GIBH Joint School of Life Sciences, The Fifth Affiliated Hospital of Guangzhou Medical University, Guangzhou Medical University, Guangzhou 510005, China; Key Laboratory of Virology and Biosafety, Wuhan Institute of Virology, Chinese Academy of Sciences, Wuhan 430071, China; Guangzhou National Laboratory, Guangzhou International Bio Island, Guangzhou 510005, China; Key Laboratory of Virology and Biosafety, Wuhan Institute of Virology, Chinese Academy of Sciences, Wuhan 430071, China

## Abstract

Chronic hepatitis B virus (HBV) persistence relies on the chromatin plasticity of covalently closed circular DNA (cccDNA), a viral minichromosome resistant to current therapies. Using proximity labeling (TurboID-dCas9), ChIP-seq and DNA pull-down assays, we identified SMARCC2—a BAF scaffolding subunit—bound to cccDNA enhancer-promoter regions (EnhⅠ/XP, CP/EnhII), where it sustains nucleosome-depleted regions (NDRs) and recruits RNA polymerase II. Genetic or pharmacological BAF inhibition compacted cccDNA chromatin, reduced histone acetylation (AcH3/AcH4), and enhanced SMC5/6-mediated silencing to suppress transcription, with the BAF ATPase inhibitor FHT-2344 reducing serum HBV DNA by 50% (*P* <.05) and intrahepatic HBV RNA by 70% (*P* <.01) without cccDNA loss, indicating epigenetic silencing. Mechanistically, BAF maintains NDRs by counteracting nucleosome retention and recruiting host transcription factors such as HNF4α. This work concludes that BAF safeguards cccDNA chromatin plasticity to enable viral persistence, and targeting BAF (e.g. FHT-2344) epigenetically silences cccDNA, offering a novel strategy for functional cure.

## Graphical Abstract

**Graphical Abstract:**
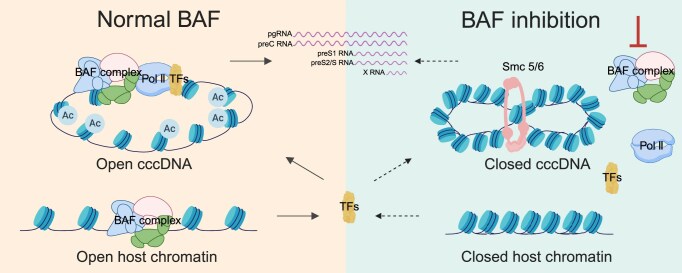

## Introduction

Hepatitis B virus (HBV), an enveloped member of the *Hepadnaviridae* family, remains a major global health challenge, with ∼254 million individuals chronically infected worldwide despite effective vaccination programs [1]. The persistence of HBV is driven by covalently closed circular DNA (cccDNA), a viral minichromosome formed in hepatocyte nuclei following virion internalization and repair of relaxed circular DNA (rcDNA) by host machinery [2, 3]. This episomal cccDNA acts as the transcriptional template for all viral RNAs, sustaining viral replication and antigen production even during antiviral therapy, thereby perpetuating chronic infection and associated pathologies such as cirrhosis and hepatocellular carcinoma [4].

The HBV minichromosome is organized as a chromatinized structure comprising cccDNA, histones, and viral proteins (HBx, HBc), which collectively regulate its transcriptional activity [5]. Its architecture includes four viral promoters [Core (CP), pre-S1 (SP1), pre-S2(SP2), X(XP)] and two enhancers (Enhancer I and II) that recruit host transcription factors (e.g. HNF4α, Sp1) and RNA polymerase II to initiate viral gene expression [6]. High-resolution epigenomic studies have identified nucleosome-depleted regions (NDRs) at these regulatory elements, which facilitate transcriptional activation [7]. CP/Enhancer II and Enhancer I/XP exhibit dynamic chromatin accessibility, functioning as nucleosome occupancy-dependent switches to control transcription [7].

cccDNA transcriptional plasticity is tightly regulated by epigenetic mechanisms. DNA methyltransferases (DNMTs) silence transcription through CpG hypermethylation [8], while histone acetyltransferases (HATs; e.g. p300/CBP) enhance activity via histone acetylation-mediated chromatin relaxation [9]. Conversely, histone deacetylases (HDACs) and methyltransferases (e.g. SETDB1) suppress transcription by promoting chromatin compaction. By recruiting host epigenetic modifiers (e.g. PCAF, HDAC1), HBx maintains a balance between activating (H3K4me3/acetylation) and repressive (H3K9me3) histone marks on the cccDNA, thereby facilitating dynamic switching between transcriptionally active and silenced states [10]. Recent research has revealed that targeted destabilization of nucleosomal architecture, with particular emphasis on the XP regulatory region, effectively suppresses both transcriptional activity and replication competence of HBV [11]. However, the spatiotemporal coordination of chromatin remodelers (e.g. BAF complex) and the maintenance of NDRs remain unresolved.

The mammalian switch defective/sucrose non-fermentable (SWI/SNF, also called BAF) complex, an ATP-dependent chromatin remodeler, regulates gene expression by repositioning nucleosomes to expose DNA regulatory elements. BAF influences infections such as human immunodeficiency virus and human papillomavirus by modulating host chromatin accessibility [12, 13]. In contrast, recent studies revealed a distinct mechanism in HBV infection, where protein arginine methyltransferase 5 (PRMT5) overexpression enhances BAF complex association with cccDNA [14]. However, the precise spatiotemporal localization of BAF complex components on HBV cccDNA and their mechanistic contributions to viral persistence remain to be fully elucidated. Here, we identify SWI/SNF related BAF chromatin remodeling complex subunit C2 (SMARCC2), a core BAF subunit, as a critical host factor promoting HBV replication by enhancing chromatin accessibility at cccDNA enhancers. Pharmacological inhibition of BAF ATPase activity suppresses viral transcription, underscoring its therapeutic potential. Our findings elucidate how host chromatin remodelers collaborate with viral factors to sustain cccDNA functionality, advancing strategies for HBV cure.

## Materials and methods

### Cell culture

The human hepatoma cell lines Huh7, Huh7-NTCP, HepG2, HepG2-NTCP were maintained in Dulbecco’s modified Eagle’s medium (DMEM; Life Technologies) supplemented with 10% (vol/vol) fetal bovine serum (FBS; Gibco, Thermo Fisher Scientific) and 100 U/ml penicillin/streptomycin (Life Technologies). HepAD38 cells were cultured in a DMEM/F12 (1:1) medium (Gibco) under the strict control of a tetracycline-responsive promoter. Primary human hepatocytes (PHHs) were obtained from Liver Biotechnology (Shenzhen) Co., Ltd and cultured in PHH maintenance medium (Cat #LV-WEM001, Liver Biotechnology Co., Ltd) per the manufacturer’s protocol. The source of the cells was listed in Supplementary Table S1.

### Mice

Female NCG-Fah KO Liver humanized mice at the age of 18 weeks were obtained from Gempharmatech Co., Ltd. (Nanjing, China), and maintained in specific-pathogen-free cages and provided autoclaved food and water in the Animal Centre of WHIOV. Upon receiving the mice, NTBC acidified water with a concentration of 8 mg/ml was provided for two days, followed by 2 mg/ml NTBC acidified water for three days, and no NTBC (0 mg/ml) acidified water for 10 days. This cycle of water providing was lasted during the experiment.

All animals were group-housed under a standard 12-h light/12-h dark cycle, and all experiments were performed during the light cycle. The animal experiments were approved by the Institutional Animal Care and Use Committee of Wuhan Institute of Virology, Chinese Academy of Sciences (approval number: WIVA02202403) and conducted in compliance with the Guide for the Care and Use of Laboratory Animals and national regulations.

### Production of HBV virions and infection

The HBV virions (genotype D, serotype ayw) were harvested from HepAD38 cell culture supernatants (ORC1159; aoruicell) and concentrated using 8% PEG8000. Viral titers were determined by quantitative polymerase chain reaction (PCR) of HBV-DNA. Concentrated virus was aliquoted and stored at −80°C. For cell infection, Huh7-NTCP or HepG2-NTCP cells were pretreated overnight with 2% FBS and 2.5% dimethyl sulfoxide (DMSO), then inoculated with 4% PEG8000 and HBV at a multiplicity of infection of 1000 viral genome equivalents/cell. Following viral adsorption, cells were washed three times with phosphate buffered saline (PBS) and maintained in fresh medium containing 2% FBS and 2.5% DMSO.

### Cell transfection

Cells were seeded in plates and grown to a confluence of 70% the following day. The plasmids were transfected into cells using the Lipofectamine 2000 reagent (Invitrogen), according to the manufacturer’s instructions. The small interfering RNAs (siRNAs) were transfected with Lipofectamine RNAiMAX reagent (Invitrogen), according to the manufacturer’s instructions. The sequences of siRNAs were listed in Supplementary Table S1.

### Enzyme-linked immunosorbent assay (ELISA)

HBsAg and HBeAg in infected cell supernatants were measured using commercial kits (Kehua Biotech) per manufacturer’s instructions. Plates were coated with anti-HBsAg or anti-HBeAg antibodies, incubated with samples, and detected using horseradish peroxidase (HRP)-conjugated antibodies with TMB substrate (A450). Human albumin in murine orbital serum was quantified using an ELISA kit (Abcam ab179887) to assess hepatocyte engraftment, as described [15].

### RNA extraction, northern blotting, and quantitative reverse transcription polymerase chain reaction (qRT-PCR)

Total RNA was extracted using the TRIzol reagent (Invitrogen). For northern bloting, 30 μg RNA were separated in denaturing agarose gel, transferred to nylon membrane, and hybridized with a [α-32P]dCTP-labeled HBV probe (prepared using random primer DNA labeling kit (Roche). Hybridization was performed at 65°C overnight in hybridization buffer (Invitrogen), following by sequential washed with 2 × saline sodium citrate (SSC)/0.1% sodium dodecyl sulphate (SDS; 10 min) and 0.2 × SSC/0.1% SDS (20 min). Signals were detected using a Phosphor-Imager system (Cyclon, Perkin Elmer).

For qRT-PCR, RNA was analyzed using Quant Studio 6 Flex system (Applied Biosystems) with a QuantiTect SYBR green reverse transcriptase-polymerase chain reaction kit (Qiangen). The primers for detecting HBV 3.5 kb RNAs and HBV total RNA were listed in Supplementary Table S1. β-actin served as the reference gene, and relative expression was calculated using the 2^−ΔΔCt^ method.

### HBV DNA extraction, southern blotting and qPCR

Cells were lysed in buffer [50 mM Tris–HCl (pH 7.4), 1 mM ethylenediaminetetraacetic acid, 1% NP-40] and treated with DNase I (10 mg/ml, 37°C, 30 min) to remove transfected plasmid DNA. Core-associated DNA was extracted by proteinase K/SDS digestion (55°C, 2 h), phenol-chloroform purification, and ethanol precipitation. cccDNA was isolated by Hirt extraction and treated with T5 exonuclease prior to qPCR to exclude DP-rcDNA interference. For a better hybridization and visualization of the cccDNA, the Hirt DNA was treated with heat denaturation by 88°C to denature the DP-rcDNA into ssDNA and further with EcoRI digestion to linearize the supercoiled cccDNA into a genome-length double-stranded DNA. For Southern blotting, DNA samples were resolved on 1% agarose gels, transferred to nylon membranes (GE Healthcare), and hybridized with HBV-specific probes. Signals were detected using a Cyclon Phosphor-Imager (Perkin Elmer). Extracellular HBV DNA from supernatants and mouse serum was extracted using TIANamp Blood DNA Kit (TIANGEN). The primers for detecting HBV rcDNA and HBV cccDNA were listed in Supplementary Table S1 with SYBR green master mix (Roche).

### Protein extraction and western blotting

Whole cell lysates were obtained by lysis of cells for 30 min on ice with radio-immunoprecipitation assay (RIPA) cell lysis buffer adding phenylmethylsulfonyl fluoride (ST506, Beyotime). Cell lysates were centrifuged at 14 000 × *g* for 10 min at 4°C, and the supernatants were recovered and denatured at 95°C for 10 min. After that, 20 μg proteins were loaded on sodium dodecyl sulphate–polyacrylamide gel electrophoresis (SDS–PAGE) and transferred to polyvinylidene fluoride membrane. Membranes were blocked with TBST (pH 7.4, containing 0.1% Tween-20) containing 5% skimmed milk for 1 h at room temperature, sequentially incubated with anti-sera containing primary antibodies overnight at 4°C, and the HRP-conjugated secondary antibodies for 1 h at room temperature. The antibody-bound protein was detected using a WesternBright ECL HRP kit (Advansta, K-12043-D20), imaged on the FluorChem HD2 system (Alpha Innotech) and analysed with AlphaEaseFC software (Alpha Innotech).

### Chromatin immunoprecipitation

Chromatin was prepared using the SimpleChIP^®^ Plus Enzymatic Chromatin IP Kit (Magnetic Beads, Cell Signaling Technology, 9005S). About 1 × 10^7^ cells were crosslinked with 1% formaldehyde (10 min, room temperature [RT]), quenched with 2.5 M glycine and washed with PBS. Nuclei were isolated and chromatin was digested with 0.5 μl micrococcal nuclease for 20 min at 37°C (Cell Signaling Technology, #10011), followed by sonication (Scientz JY88-IID sonicator, 95% power, 9.9 s on/2 s off, 10 cycles). The supernatants were incubated overnight at 4°C with indicated antibodies, then the complexes were captured with Protein G Magnetic Beads (2 h, 4°C), washed three times with low- and high-salt buffer, and eluted with chromatin immunoprecipitation (ChIP) Elution buffer (30 min, 65°C, 1200 rpm vortexing). The antibodies used for ChIP were listed in Supplementary Table S1. Crosslinks were reversed (5 M NaCl, proteinase K, 65°C overnight) followed by RNase treatment (1 h, 37°C). ChIP DNA was purified and analyzed by quantitative real-time PCR (qPCR) using HBV or GAPDH specific primers, or by Next generation sequence (NGS) sequencing. The specific primers for ChIP-qPCR were listed in Supplementary Table S1.

### Next generation sequence

The ChIP DNA from Huh7 cells transfected with prcccDNA, pCre and pCMV-SMARCC2-3 × Flag were analyzed by NGS. All the next procedures were finished by Sangon Biotech (Shanghai) Co., Ltd. NGS^®^ Max Up II DNA Library Prep Kit for Illumina^®^ (YEASEN, Shanghai, China)was used for library preparations. Briefly, Endprep enzyme was added to repair end and 3′ end A tail ligation. Then adaptor was ligated by enhancer and Fast T4 DNA ligase. Index primer was added by PCR and the amplified product about 400 bp was selected by DNA selection beads. The library concentration and size were confirmed by Qubit 4.0 (Thermo, Waltham, USA) and 2% agarose gel electrophoresis respectively. Then the libraries were pooled and loaded on Novaseq 6000 (Illumina, San Diego, USA)/DNBseq-T7 (BGI, Shenzhen, China) sequencer by 2 × 150 bp paired end sequence kit according to the manufacturer’s instructions.

Raw reads containing adaptor sequences and those with ambiguous or low-quality bases at the beginning or end were trimmed using Trimmomatic (0.39) [16]. The qualified reads from each sample were aligned to the assembled reference genome using BWA (0.7.17) [17] with default parameters. MACS2 (V2.1.1) [18] was used to call peaks with the *P*-value <1e-5 and annotated to the gene functional region. Functional enrichment analysis was finished by the peak annotated genes using top gene ontology (GO [2.36.0]) for GO terms and clusterProfiler (3.12.0) [19] for kyoto encyclopedia of genes and genomes (KEGG) pathways, respectively. Terms or pathways with *P*-values < 0.05 were regarded as significantly enriched.

### DNA-pulldown assay

Huh7 cells were transfected with pSMARCC2-3 × flag plasmid for 48 h. Nuclear extracts were prepared and incubated with biotinylated DNA probes (Supplementary Table S1) conjugated to streptavidin magnetic beads (Dynabeads M280). Then, 100 pmol of 5′-biotin-labeled probes (generated by PCR or oligonucleotide annealing) were bound to 200 μl beads (Dynabeads, M280), then incubated with 800 μg nuclear extracts and 20 μg herring sperm DNA (Sigma) at 4°C. Bound proteins were eluted, separated by 10% SDS–PAGE, and detected by immunoblotting analysis with a Mouse anti-Flag antibody.

### Bulk RNA-seq analysis

PHHs infected with HBV were treated with FHT-1015 for 12 days. Total RNA was extracted and quality-checked before library preparation. Poly(A)+ RNA was enriched from 1 μg total RNA using VAHTS mRNA Capture Beads (Vazyme, N401), followed by complementary DNA (cDNA) synthesis and library construction with VAHTS Universal V8 RNA-seq Library Prep Kit (Vazyme, NR605). Libraries were sequenced on an Illumina platform (Guangzhou LAB).

For bioinformatic analysis, raw reads were quality-filtered using FASTP (v0.23.4) [20] and aligned to the human reference genome (GRCh38, ENSEMBL 111) with STAR (v2.7.11b) [21]. Gene expression quantification was performed using RSEM (v1.3.1) [22]. Differential expression between FHT-1015-treated and control samples was analyzed with DESeq2 (v1.42.1) [23], applying thresholds of adjusted *P*-value < 0.05 (Benjamini–Hochberg) and |log_2_FC| >1. HBV RNA was quantified by aligning reads to NC_003977.2.

### ATAC-seq data analysis

Cultured cells (50 000 per sample) processed immediately for nuclei isolation and transposase treatment using the High-Sensitivity Open Chromatin Profile Kit (Novoprotein, N248). All libraries were sequenced using SURFSeq 5000 sequencer with PE150 reads.

Raw sequencing data underwent adapter trimming and sequence quality filtering employing fastp [24] (v0.23.4). Filtered reads were aligned to the Homo sapiens reference genome GRCm38 (ENSEMBL assembly) using Bowtie2 (v2.5.2) [25] with parameters optimized for chromatin accessibility data(–very-sensitive-local –no-unal –no-mixed –no-discordant -I 10 -X 700). Duplicated reads were discarded; high quality reads were extracted use samtools (v1.19) [26] (-q 2 -F 1804 -f 2). Peak calling was conducted with MACS2 [18] under parameters –keep-dup all –gsize hs. Differential chromatin accessibility was quantified using DiffBind (v3.12.0 FDR ≤ 0.05, |log_2_FC|>1). Genomic peak annotation was performed via ChIPseeker (v1.38.0) [27] to define transcription start site (TSS)-proximal and distal regulatory elements. Normalized signal tracks (bin size = 50 bp) were generated using bamCoverage (deepTools v3.5.4 [27] to compute bins per million mapped reads. For HBV integration analyses, signal normalization was adjusted to reads per genome coverage using –binSize 1 –smoothLength 21. Genome-wide chromatin accessibility profiles were visualized using the Integrative Genomics Viewer.

### RNA-seq and ATAC-seq library preparation and sequencing

For RNA-seq, mRNA was captured by VAHTS mRNA Capture Beads (Vazyme, N401) from 1 μg total RNA. RNA fragmentation, cDNA synthesis and library preparation were accomplished by VAHTS Universal V8 RNA-seq Library Prep Kit for Illumina (Vazyme, NR605) according to the manufacturer’s instructions.

For ATAC-seq, 50 000 cells were collected freshly to ensure highly viability. Nuclei exposure and transposase reaction were completed immediately after cell preparation. Fragmented DNA were purified for ATAC library preparation using the High-Sensitivity Open Chromatin Profile Kit for Illumina (Novoprotein, N248).

All libraries were sequenced using SURFSeq 5000 sequencer with PE150 reads

### FHT-2344 treatment and HBV infection of NCG-Fah KO liver humanized mice

Female mice were randomly divided into the control (*n* = 4) and treatment groups (*n* = 4). For HBV infection, the mice received 200 μl of HBV from HepAD38 cells (5 × 10^9^ VGE) intravenously. The BAF ATPase inhibitor FHT-2344 (HY-149458) was dissolved in DMSO to a concentration of 50 mg/ml. Then, 10% of the reconstituted FHT-2344 was added to corn oil immediately before gavage administration and administered at 20 mg/kg daily. Control (*n* = 4) and FHT-2344 (*n* = 4) treatments began 7 days after HBV infection and continued for 2 weeks. The liver tissues and whole blood were collected at the indicated time points. The liver tissues were submitted to Hubei BIOSSCI Biotechnology Co., Ltd. for quality assessment and immunofluorescence assays.

### Statistical analyses

Statistical analyses were performed with GraphPad Prism (GraphPad Software). Continuous variables are reported as means ± the standard deviations (SD) unless indicated otherwise. Pairs of groups were compared by using a two-tailed Student’s *t*-test (normal distribution data). For multiple groups comparisons, a one-way analysis of variance (ANOVA) was conducted. *P *<.05 was considered statistically significant.

## Results

### Identification of HBV core promoter associated host factors in HBV infection

**Figure 1.**
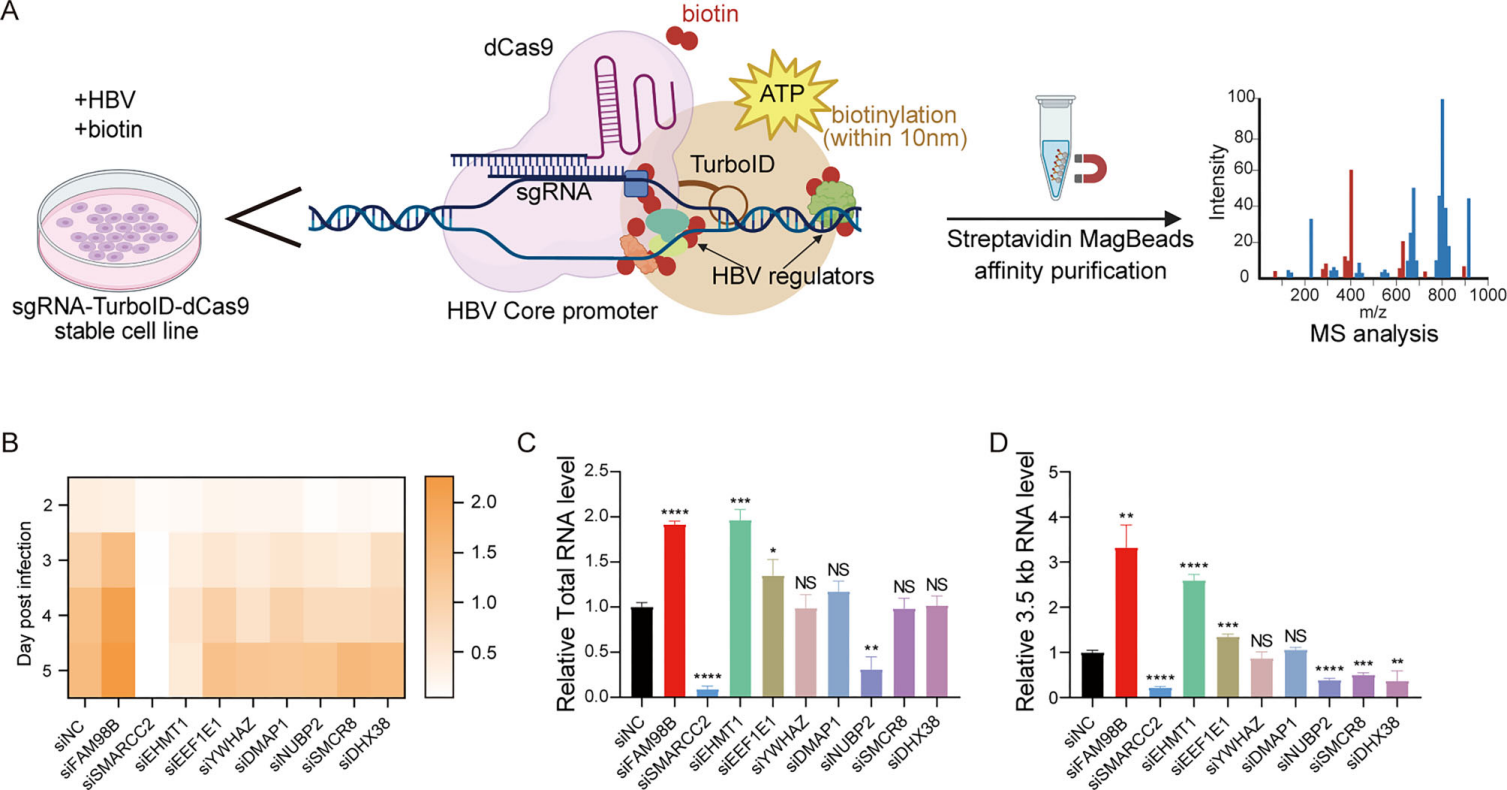
Identification of HBV CP related host factors. (**A**) System design. A TurboID-dCas9 fusion protein, guided by sgRNAs to the HBV CP region, biotinylates proximal proteins (∼10 nm radius) for subsequent identification. (**B–D**) Candidate host factor validation. Huh7-NTCP cells were transfected with gene-specific siRNAs (or siNC control) for 48 h, infected with HBV (1000 VGE/cell), and analyzed. (**B**) Time-course HBeAg secretion (ELISA). (**C, D**) Intrahepatic HBV RNA at 5 dpi: (**C**) total HBV RNA and (**D**) 3.5 kb RNAs (qRT-PCR). Data presentation: Mean ± SD from three independent biological replicates. Statistics: one-way ANOVA with Tukey’s test (*P <.05, **P <.01, ***P <.001, ****P <.0001). All qRT-PCR data were normalized to actin. The results from one representative experiment are shown. Experiments were repeated three times.

TurboID-dCas9 technology [28] was developed to elucidate host factors associated temporally or constitutively with HBV cccDNA. Briefly, a fusion protein was constructed by linking catalytically inactive dCas9 (a Cas9 mutant retaining guide RNA and target DNA binding capability but lacking endonuclease activity) to the biotin ligase TurboID. This TurboID-dCas9 fusion was then targeted to cccDNA using single guide RNAs (sgRNAs) complementary to the viral core promoter (CP) region (nt 1636–1852), a region critical for viral gene expression. Following the addition of biotin to the cell culture medium, proteins in proximity to this site were biotinylated by TurboID (Fig. 1A). Delivery of Cas9 together with four sgRNAs directed at the CP region led to a significant reduction in supernatant levels of HBeAg, HBsAg, and HBV DNA in HepAD38 cells (Supplementary Fig. S1A–D). A stable Huh7-NTCP cell line expressing sgRNA1 (nearby CP, nt 1618–1637) TurboID-dCas9 was used for subsequent proximity-dependent proteomic profiling for its superior biotinylation efficiency (Supplementary Fig. S1E and F). To mitigate nonspecific labeling, we compared the biotinylated proteins in sgRNA1 turboID-dCas9 cell with and without HBV infection at 3 and 5 days post infection (dpi). Proteomic analysis identified 135 proteins significantly enriched (fold change ≥ 1.5, *P* <.05) during infection (Supplementary Table S2), including known cccDNA regulators such as tyrosyl–DNA phosphodiesterase 2 (TDP2), proliferating cell nuclear antigen (PCNA), heterogeneous nuclear ribonucleoprotein K (HNRNPK), C-terminal binding protein 1 (CTBP1), POLR2C (RNA Polymerase II Subunit C) [29–33]. Interestingly, key repair factors such as TDP2 and PCNA were identified, implicating host factors that bind to HBV intermediates such as rcDNA and protein-free rcDNA.

From the identified proteins, nine candidates (family with sequence similarity 98, member B [FAM98B], SMARCC2, euchromatic histone-lysine N-methyltransferase 1 [EHMT1], eukaryotic translation elongation factor 1 epsilon 1 [EEF1E1], tyrosine and tryptophan hydroxylase activators zeta [YWHAZ], methyltransferase 1 associated protein 1 [DMAP1], nucleotide binding protein 2 [NUBP2], Smith-magenis syndrome chromosome region candidate 8 [SMCR8] and DEAH-box helicase 38 [DHX38]) with established role in epigenetic modification or RNA transcription were selected for further functional validation. siRNA-mediated knockdown in HBV-infected Huh7-NTCP cells revealed different effect on viral replication. While most siRNAs cause a moderate decrease in HBeAg level at 3 and 4 dpi, FAM98B knockdown significantly enhanced HBeAg secretion, whereas SMARCC2 knockdown markedly suppressed it (Fig. 1B). Consistent with these findings, FAM98B knockdown significantly increased HBV total RNA and 3.5 kb RNAs level, while SMARCC2 and NUBP2 knockdown markedly reduced HBV total RNA and 3.5 kb RNAs level (Fig. 1C and D). Given SMARCC2’s role as a core component of the SWI/SNF chromatin remodeling complex—a key driver of epigenetic regulation—we further investigated its mechanistic contribution to HBV replication.

### SMARCC2 is required for HBV infection and replication

**Figure 2.**
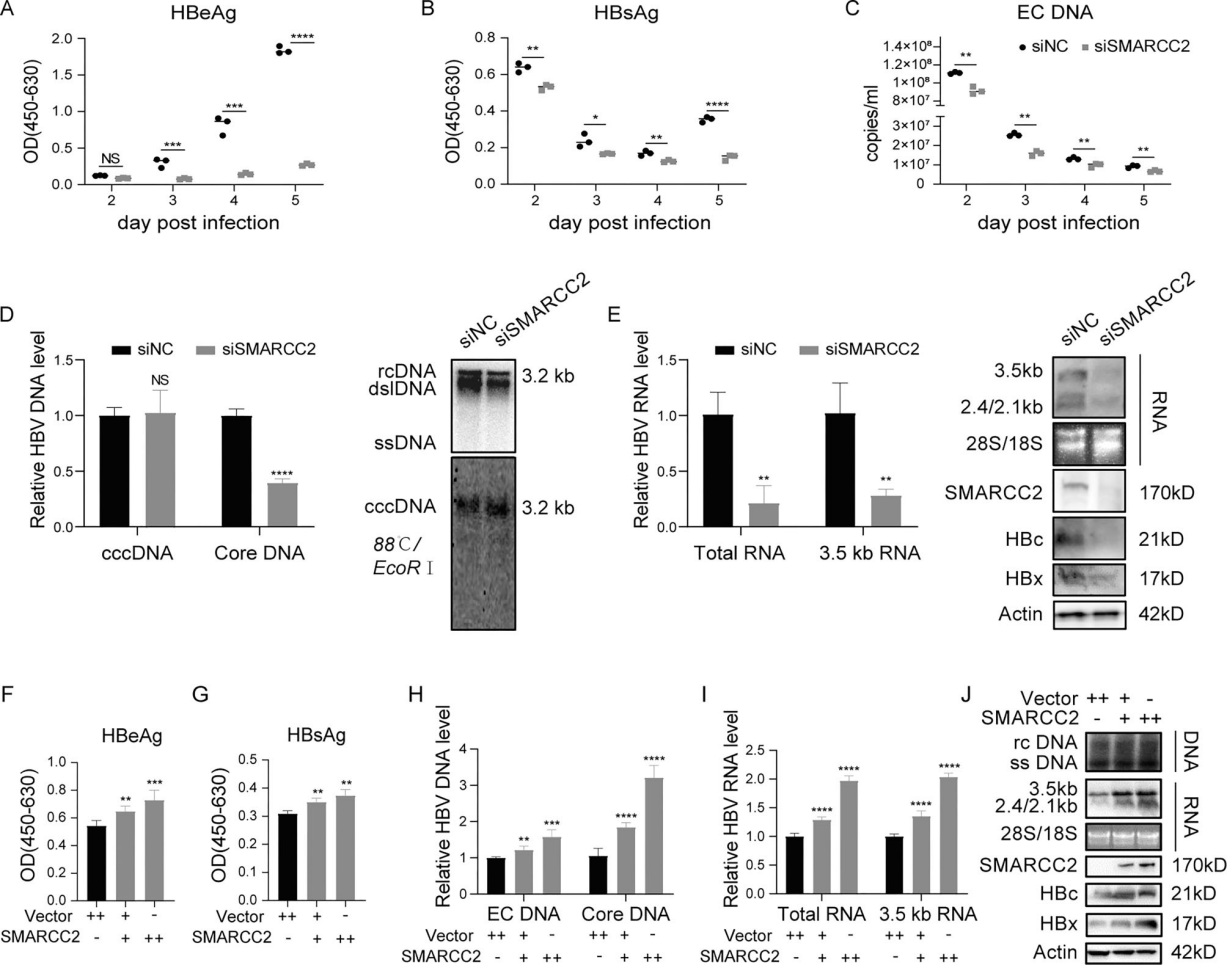
SMARCC2 regulates HBV infection and replication in hepatoma cells. (**A–E**) SMARCC2 knockdown inhibits HBV infection in Huh7-NTCP cells. Huh7-NTCP cells were transfected with SMARCC2-targeting siRNA (siSMARCC2) or negative control siRNA (siNC) for 48 h, then infected with HBV at a multiplicity of 1000 viral genome equivalents (VGE) per cell for 16 h. Following three PBS washes, cells were maintained in complete medium. (**A**) HBeAg and (**B**) HBsAg levels in daily collected supernatants were quantified by ELISA. (**C**) Extracellular HBV DNA (EC DNA) was measured by quantitative PCR. At 5 dpi, (**D**) Intracellular HBV DNA forms (cccDNA and core-associated DNA [Core DNA]) were analyzed by quantitative PCR and Southern blotting. (**E**) HBV RNA species (total RNA and 3.5 kb RNAs) were detected by qRT-PCR and northern blotting. SMARCC2 knockdown efficiency and viral protein (HBc and HBx) expression were assessed by western blotting (WB). (**F–J**) SMARCC2 overexpression enhances HBV replication and transcription. Huh7 cells in 12-well plates were cotransfected with 1 μg HBV replication-competent plasmid (pSM2) and either 1 μg empty vector or pSMARCC2 expression plasmid. Cells and supernatants were harvested at 48 h posttransfection. (**F**) HBeAg and (**G**) HBsAg secretion were quantified by ELISA. (**H**) Extracellular HBV DNA (EC DNA) and core-associated HBV DNA (Core DNA) was quantified by quantitative PCR. (**I**) HBV RNA species (total RNA and 3.5 kb RNAs) were detected by qRT-PCR. (**J**) SMARCC2 overexpression and viral markers were examined by WB (proteins), northern blotting (RNA), and Southern blotting (DNA). Data represent mean ± SD from three independent biological replicates. Statistical analyses were performed using two-tailed Student’s *t*-test (for two-group comparisons) or one-way ANOVA with Tukey’s post *hoc test* (for multiple comparisons). Significance levels: *P <.05, **P <.01, ***P <.001, ****P <.0001. All qRT-PCR data were normalized to actin. The results from one representative experiment are shown. Experiments were repeated at least three times.

To define the mechanism of SMARCC2 in HBV replication, we first evaluated its necessity using siRNA-mediated knockdown. Although CRISPR-Cas9 failed to generate SMARCC2 knockout mutant cells, siRNA-mediated knockdown achieved > 70% efficiency without inducing cytotoxicity (Supplementary Fig. S1G). Meanwhile, SMARCC2 knockdown did not affect cell viability or differentiation in Huh7-NTCP cells within 7 days post transfection (Supplementary Figs S1H and I). SMARCC2 knockdown persistently reduced HBeAg, HBsAg, and extracellular HBV DNA levels from 2 dpi, with marked reductions at 5 dpi (HBeAg: 90%, *P* <.0001, HBsAg: 60%, *P* <.0001, HBV DNA: 50%, *P* <.01) in culture supernatants of HBV infected Huh7-NTCP (Fig. 2A–C). Intriguingly, SMARCC2 knockdown did not alter HBV cccDNA levels but decreased the level of core-associated HBV DNA, HBV total RNA, 3.5 kb RNAs, as well as HBc and HBx protein (Fig. 2D and E), indicating that SMARCC2 regulates viral transcription rather than early replication steps.

HBV plasmid transient-transfection system and Cre mediated HBV recombinant cccDNA system were used to dissect this further. SMARCC2 overexpression in Huh7 cells transfected with HBV replication plasmid pSM2 (under regulation of HBV authentic promoter) enhanced HBeAg (1.3-fold, *P* <.001; Fig. 2F), HBsAg (1.3-fold, *P* <.01; Fig. 2G), extracellular HBV DNA (1.5 fold, *P* <.001; Fig. 2H), core-associated HBV DNA (3-fold, *P* <.0001; Fig. 2H), intracellular HBV RNA (2-fold, *P* <.0001; Fig. 2I) and HBc, HBx proteins (Fig. 2J) in a dose-dependent manner. Similar results were obtained in the Cre mediated HBV recombinant cccDNA system, SMARCC2 knockdown decreased HBeAg, HBsAg and HBV RNAs level (Supplementary Fig. S2A–C), while its overexpression increased HBeAg, HBsAg and HBV RNAs level (Supplementary Fig. S2D–F).

To exclude cell type-specific effects, we validated these findings in HepG2-NTCP and HepG2 cells. SMARCC2 knockdown consistently inhibit HBV infection and replication in HepG2-NTCP (Supplementary Fig. S3A–E), while its overexpression promotes HBV replication from pSM2 in HepG2 (Supplementary Fig. S3F–J).

### SMARCC2 enhances HBV CP and XP activity as a cBAF complex

**Figure 3.**
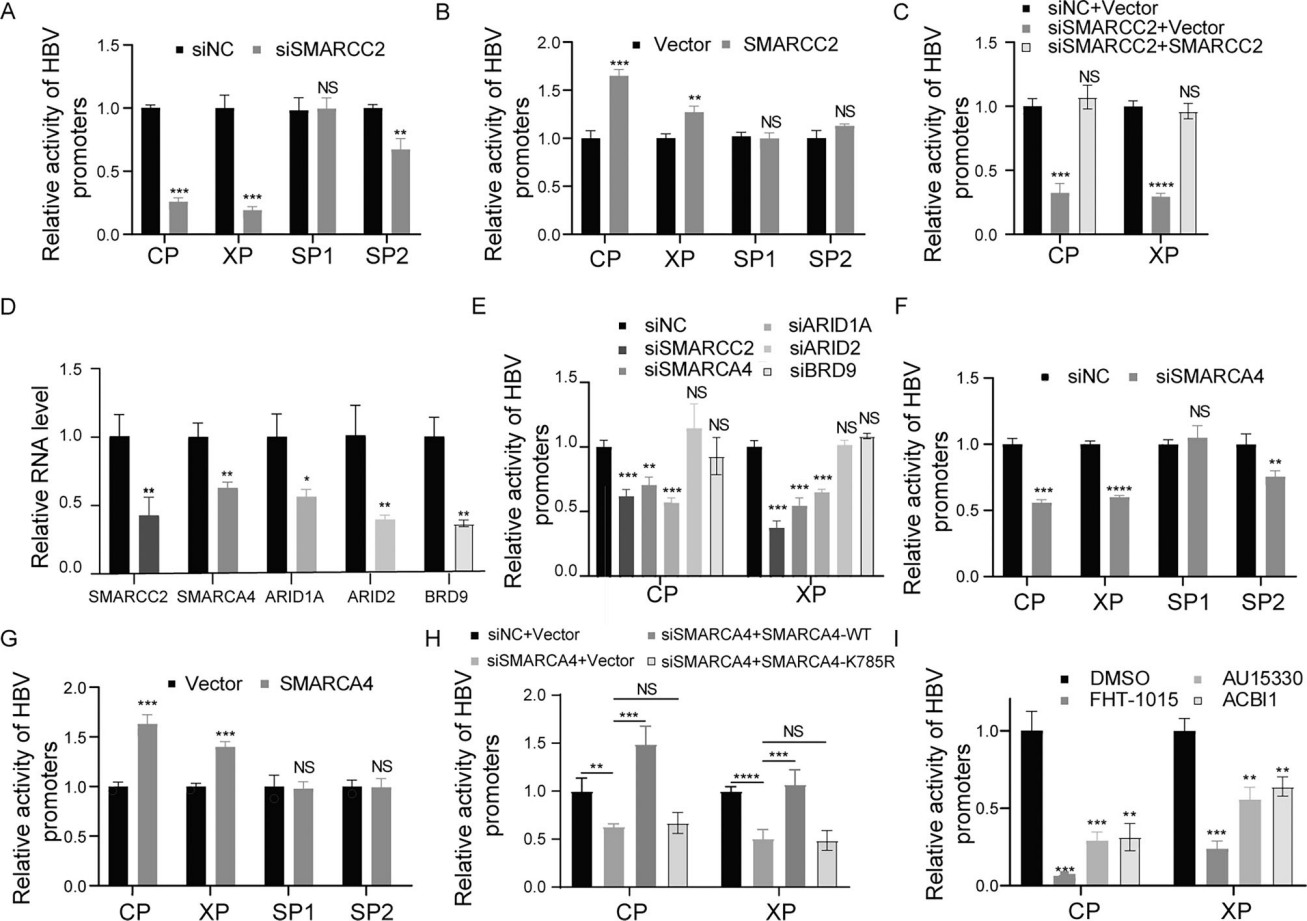
SMARCC2 mediates cBAF-dependent enhancement of HBV core (CP) and X (XP) promoter activity. (**A, B**) Huh7 cells were cotransfected with HBV promoter (CP, XP, SP1, SP2) luciferase reporters and either (**A**) siSMARCC2 or (**B**) SMARCC2 overexpression plasmid (pSMARCC2). Luciferase activity was measured at 48 hpt. (**C**) Huh7 cells were cotransfected with control (siNC or vector) and SMARCC2-modulating constructs (siSMARCC2 or pSMARCC2). Activity was measured at 48 hpt. (**D**) Cells were transfected with siRNAs targeting SMARCC2, SMARCA4, ARID1A, ARID2, or BRD9. BAF subunit knockdown efficiency were measured by qRT-PCR. (**E**) HBV CP or XP reporter assays with BAF subunit knockdown. Huh7 cells were cotransfected with promoter reporters and individual siRNAs [as in panel (D)]. Luciferase activity was measured at 48 hpt. (**F, G**) Promoter specificity analysis: HBV reporters were cotransfected with (**F**) siSMARCA4 or (**G**) pSMARCA4. (**H**) Empty vector, pSMARCA4-WT or pSMARCA4-K785R and reporter plasmids were transfected to SMARCA4 knowdown Huh7 cells, and the CP or XP reporter assays were then performed. (**I**) Huh7 cells transfected with CP or XP reporters were treated (2 hpt) with ATPase inhibitor FHT-1015 or PROTAC degraders (AU15330, ACBI1). Activity was measured at 48 hpt. Data represent mean ± SD from three independent biological replicates. Statistical analyses were performed using two-tailed Student’s *t*-test (for two-group comparisons) or one-way ANOVA with Tukey’s *post hoc* test (for multiple comparisons). Significance levels: *P <.05, **P <.01, ***P <.001, ****P <.0001. The results from one representative experiment are shown. Experiments were repeated at least three times.

To define the mechanism underlying SMARCC2-mediated transcriptional regulation of HBV, we performed luciferase reporter assays targeting the four major HBV promoters (CP, XP, SP1, SP2). SMARCC2 knockdown in Huh7 cells significantly suppressed HBV CP (60% reduction, *P* <.001) and XP (70% reduction, *P* <.001) activities, with a modest suppression effect on the SP2 promoter (25% reduction, *P* <.01), while SP1 remained unaffected (Fig. 3A). Conversely, SMARCC2 overexpression markedly enhanced CP (1.75-fold, *P* <.001) and XP (1.25-fold, *P* <.01) activity but not SP1 and SP2 (Fig. 3B). Critically SMARCC2 overexpression rescued CP and XP inhibition caused by its knockdown (Fig. 3C), confirming its direct role in regulating these promoters.

As SMARCC2 is a scaffolding subunit essential for BAF complex assembly, we next probed which BAF subtype—canonical (cBAF), polybromo-associated (pBAF), or noncanonical (ncBAF)—mediates this regulation. To distinguish between subtypes, we targeted signature subunits: SWI/SNF related BAF chromatin remodeling complex subunit ATPase 4 (SMARCA4, common to all BAF complexes), AT-rich interaction domain 1A (ARID1A, cBAF-specific subunit), AT-rich interaction domain 2 (ARID2, pBAF-specific subunit) and bromodomain containing 9 (BRD9, ncBAF-specific subunit) (Fig. 3D). Subtype-specific siRNA knockdown revealed that SMARCA4 and ARID1A depletion recapitulated the CP and XP suppression seen with SMARCC2 depletion (CP: 30%–40% ↓, XP: 50%–60% ↓; *P* <.01), whereas ARID2 or BRD9 silencing had no effect (Fig. 3E). Similar with SMARCC2, knockdown or overexpression of SMARCA4 modulated the activity of CP and XP but not SP1 and SP2 (Fig. 3F–G). Expression of the wild-type SMARCA4 was sufficient to rescue the CP and XP inhibition following its knockdown, whereas the catalytically dead mutant (K785R) failed to do so (Fig. 3H). Pharmacological disruption of SMARCA2/4, the ATPase catalytic subunits of BAF complexes, using either the ATPase inhibitor FHT-1015 or PROTAC degraders (AU15330, ACBI1), abolished CP and XP activity (Fig. 3I) (34–36), implicating BAF enzymatic function in promoter activation. These results pinpoint the cBAF complex as the mediator of SMARCC2-driven promoter activation.

### Pharmacological inhibition of the BAF complex inhibits HBV replication at the transcriptional level

**Figure 4.**
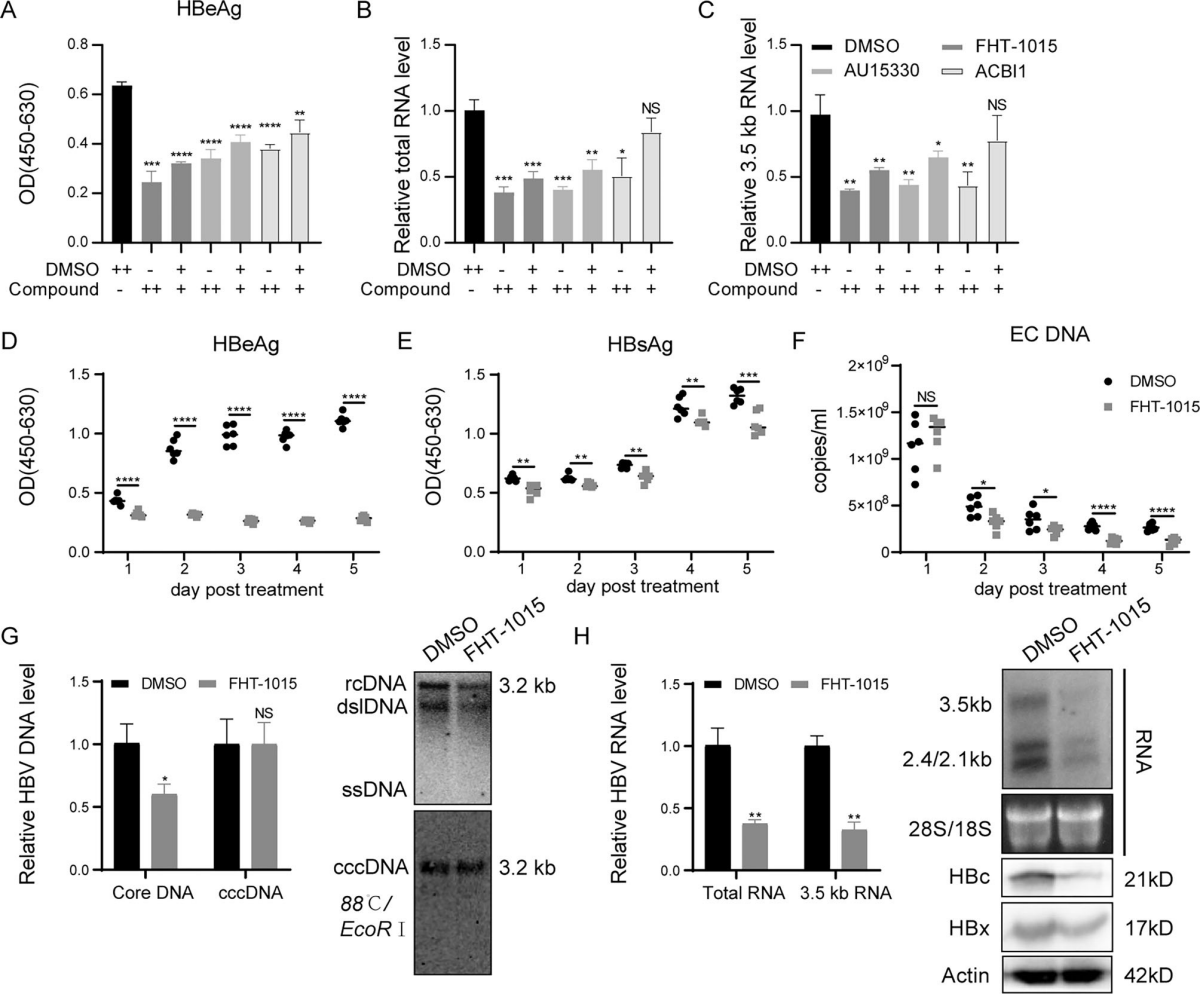
BAF ATPase inhibitors suppress HBV transcription in hepatoma cells. (**A–C**) Huh7-NTCP cells were infected with HBV virions (1000 VGE/cell, derived from HepAD38) for 5 days in the presence of DMSO, the BAF ATPase inhibitor FHT-1015, or the PROTAC degrader AU15330 or ACBI1. Supernatants were replaced every 2 days, and cells/supernatants were harvested at 5 dpi. (**A**) HBeAg levels were quantified by ELISA, (**B**) HBV total RNA and (**C**) 3.5 kb RNAs were measured by qRT-PCR. (**D–H**) HepG2-NTCP cells were infected with HBV (1000 VGE/cell) for 3 days then treated with DMSO or FHT-1015 for 5 days. (**D**) HBeAg, (**E**) HBsAg and (**F**) extracellular HBV DNA (EC DNA) in daily collected supernatants were quantified by ELISA or qPCR. (**G**) Intracellular HBV DNA forms (cccDNA and core-associated DNA [Core DNA]) were analyzed by quantitative PCR and Southern blotting. (**H**) HBV RNA species (total RNA and 3.5 kb RNAs) were detected by qRT-PCR and northern blotting. Viral protein (HBc and HBx) expression were assessed by WB. Data represent mean ± SD from at least three independent biological replicates. Statistical analyses were performed using two-tailed Student’s *t*-test or one-way ANOVA with Tukey’s post hoc test (for multiple comparisons). Significance levels: *P <.05, **P <.01, ***P <.001, ****P <.0001. All qRT-PCR data were normalized to actin.

We next investigated the regulatory role of the BAF complex in HBV infection. Huh7-NTCP cells infected with HBV (1000 VGE/cell) were treated with three characterized BAF inhibitors: FHT-1015 (1 and 2 μM), AU15330 (1 and 2 μM), and ACBI1 (1 and 2 μM). All compounds dose-dependently suppressed HBV replication (Fig. 4A–C). At 5 dpi, 2 μM concentrations significantly reduced HBeAg secretion by 40%–50% (*P* <.0001), intracellular total HBV RNA by 50%–60% (*P* <.01), and 3.5 kb RNAs by 40%–60% (*P* <.001) compared to DMSO controls. Notably, FHT-1015 exhibited the strongest inhibitory effect at 1 μM, while ACBI1 showed no significant activity at this concentration, suggesting divergent potency among BAF-targeting compounds. These results mirror SMARCC2 knockdown phenotypes, confirming BAF’s critical role in post-entry transcriptional regulation of HBV.

To further confirm BAF’s transcriptional regulation of cccDNA, FHT-1015 treatment was performed at 3 days post HBV infection, a timepoint when the cccDNA pool stabilizes [37, 38], with HBV markers monitored from 1–5 days post treatment. WST-1 assay confirmed no effect of FHT-1015 on cell viability (Supplementary Fig. S4A). Hepatocyte differentiation status, assessed by morphology and mRNA levels of markers (ASGPR, Albumin, AFP, CYP3A4), was similar between DMSO and FHT-1015 treated cells (Supplementary Fig. S4B). By day 5 post-treatment, FHT-1015 significantly reduced supernatant HBeAg (80%, *P* <.0001), HBsAg (30%, *P* <.001) and extracellular HBV DNA level (60%, *P* <.0001; Fig. 4D–F). Intracellularly, it reduced core-associated HBV DNA (45%, *P* <.05), HBV total RNA (60%, *P* <.01), 3.5 kb RNAs (65%, *P* <.01), as well as HBc and HBx protein (Fig. 4G and H). Critically, the cccDNA level and the stability of HBV RNAs remained unchanged (Fig. 4G and Supplementary Fig. S4C), confirming that pharmacological inhibition of BAF complex inhibits HBV replication at the transcriptional level.

To further rule out genotype-specific effects, we examined the impact of SMARCC2 on HBV replication across multiple genotypes (A, B, C, E, F, H, and J; Supplementary Fig. S5). The results showed a consistent pattern: SMARCC2 overexpression enhanced viral transcription, while the BAF complex inhibitor FHT-1015 suppressed it, irrespective of the genotype. Notably, this functional conservation strongly indicates that the BAF complex regulates HBV transcription in a genotype-independent manner. Furthermore, we examined the effect of BAF modulation on integrated HBV sequences in HepG2.2.15, Hep3B and PLC/PRF/5 cells. To specifically assess transcription from integrated viral DNA without interference from episomal cccDNA, HepG2.2.15cells were treated with 3TC, a nucleoside analog that inhibits reverse transcription and prevents de novo cccDNA formation. Under these conditions, all detected HBV RNAs derived from the integrated viral genome. Treatment with FHT-1015 suppressed HBV RNA and protein expression, indicating that the BAF complex plays a conserved role in viral transcription from integrated sequences in the HepG2.2.15 cell model (Supplementary Fig. S6A–D). Similarly, in Hep3B (Supplementary Fig. S6E and F) and PLC/PRF/5 (Supplementary Fig. S6G and H) cells, FHT-1015 treatment resulted in a modest but statistically significant decrease in both HBs RNA and secreted HBsAg levels. Together, these findings suggest that the BAF complex regulates transcription from integrated HBV sequences in the tested models.

### Pharmacological inhibition of BAF complex reduces HBV infection in PHHs

**Figure 5.**
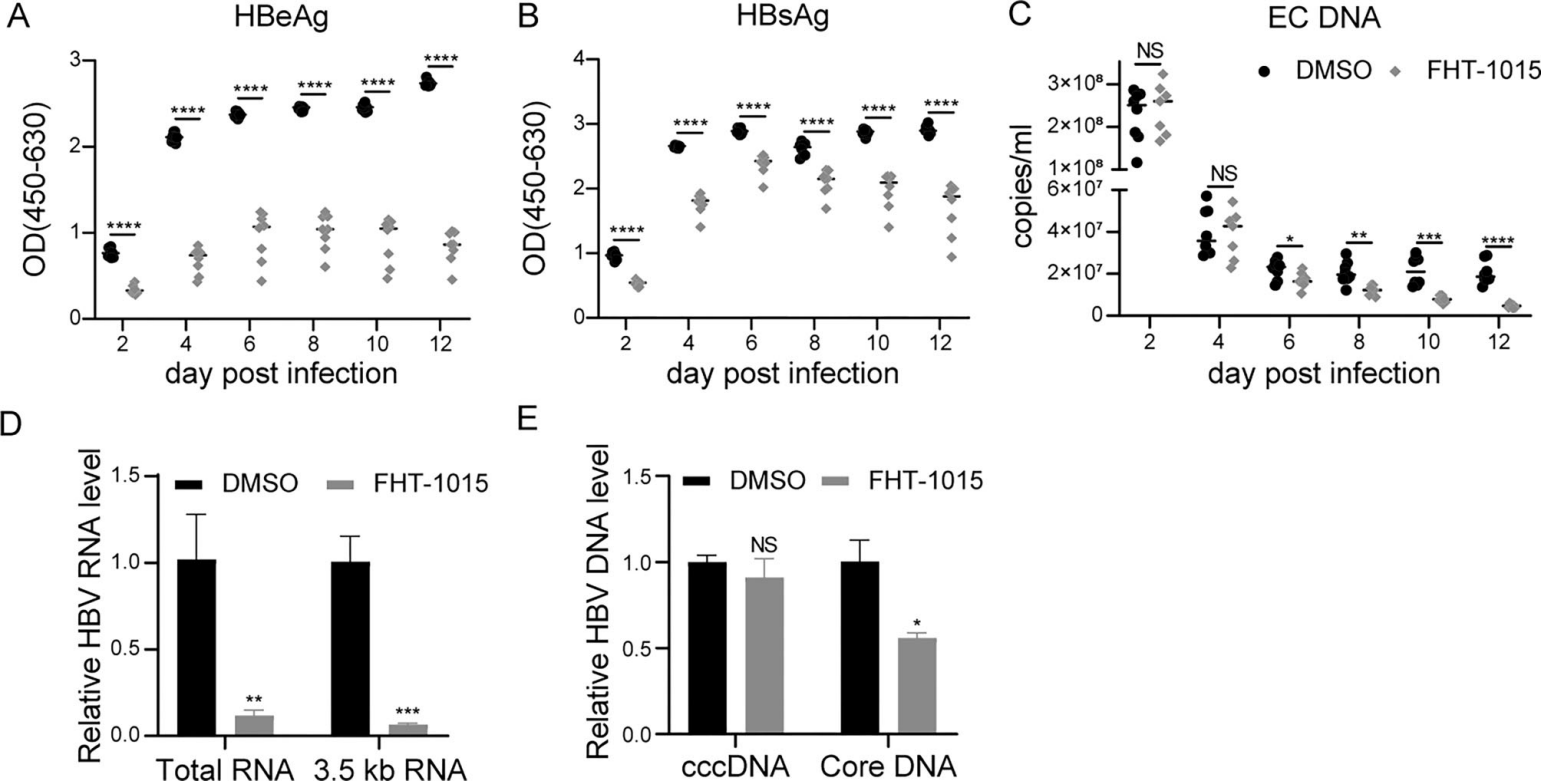
BAF ATPase inhibitors suppress HBV infection in PHHs. (**A–E**) PHHs were infected with HBV (1000 VGE/cell, HepAD38-derived) for 12 days with DMSO or FHT-1015 treatment. (**A**) HBeAg and (**B**) HBsAg in supernatants were assessed by ELISA at 2, 4, 6, 8, 10, and 12 dpi. (**C**) Extracellular HBV DNA (EC DNA) was quantified by qPCR at the same time points. (**D**) Intracellular HBV RNA and (**E**) HBV DNA forms (cccDNA and core-associated DNA [Core DNA]) were analyzed by qRT-PCR and qPCR at 12 dpi. Data represent mean ± SD from at least three independent biological replicates. Statistical analyses were performed using one-way ANOVA with Tukey’s *post hoc* test (for multiple comparisons). Significance levels: *P <.05, **P <.01, ***P <.001, ****P <.0001. All qRT-PCR data were normalized to actin.

To validate findings in a physiologically relevant system, we infected PHHs with HBV (1000 VGE/cell), and treated them with FHT-1015 (2 μM) starting at 24 hpi. Longitudinal analysis revealed progressive declines in HBeAg and HBsAg secretion (2–12 dpi, *P* <.001; Fig. 5A and B), accompanied by a reduction in extracellular HBV DNA (6–12 dpi, *P* <.05–.001; Fig. 5C). Intracellular viral RNA (total RNA: 85% ↓; 3.5 kb RNAs: 90% ↓, *P* <.001) and core-associated HBV DNA (50% ↓, *P* <.05) were suppressed at 12 dpi, while cccDNA levels remained unchanged (Fig. 5D and E). This mirrors SMARCC2 depletion phenotypes, reinforcing BAF complex’s selective role in regulating cccDNA transcription rather than its stability.

### The BAF complex directly binds and remodels chromatin accessibility at HBV regulatory elements

**Figure 6.**
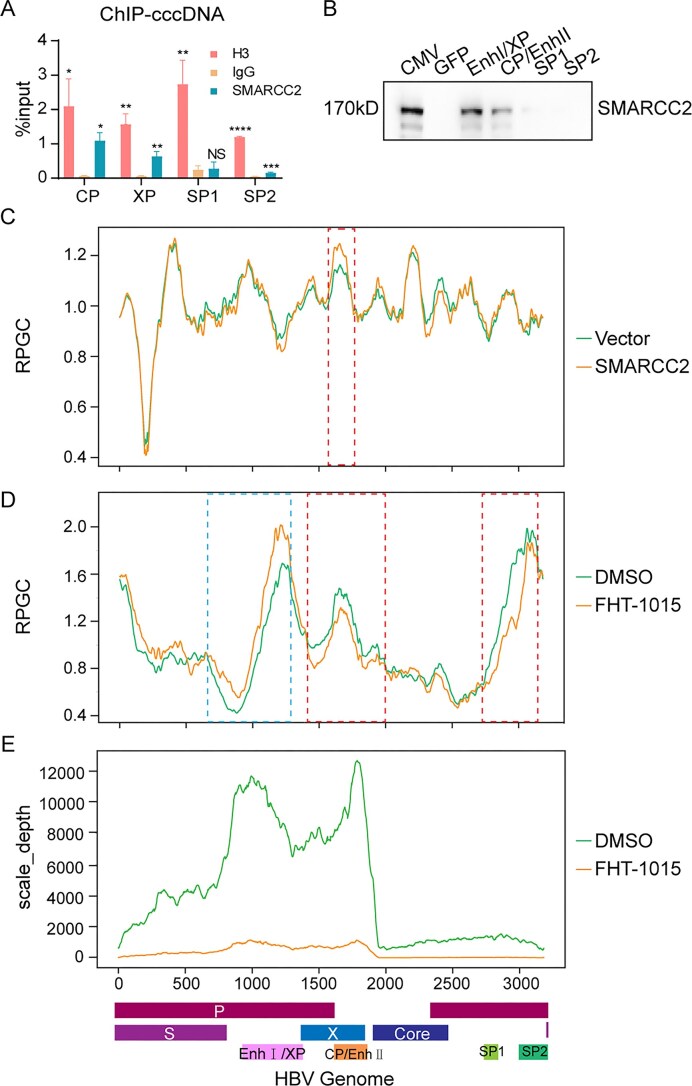
BAF complex binds to HBV regulatory regions and enhances chromatin accessibility. (**A**) ChIP assay of HBV-infected HepG2-NTCP cells (5 dpi) using anti-SMARCC2, anti-H3, or IgG antibodies. qPCR analysis shows SMARCC2 binding at HBV cccDNA regions (CP/EnhII and EnhⅠ/XP) relative to input. Data represent mean ± SD (*n* = 3). (**B**) DNA-pulldown assay with HBV regulatory probes matched to CP/EnhII, EnhⅠ/XP, SP1 and SP2 promoter incubated with nuclear extracts from SMARCC2-transfected Huh7 cells. CMV-TATA and GFP served as positive and negative controls, respectively. (**C**) ATAC-seq analysis of Huh7 cells co-transfected with prcccDNA, pCre, and pSMARCC2 (harvested at 48 hpt). Representative tracks show increased chromatin accessibility at HBV CP region upon SMARCC2 overexpression (*n* = 2). (**D, E**) PHHs were infected with HBV (1000 VGE/cell, HepAD38-derived) for 12 days and treated with DMSO or FHT-1015. (**D**) ATAC-seq tracks demonstrate FHT-1015-mediated reduction in chromatin accessibility at HBV CP, SP1, and SP2 regions and inrease in chromatin accessibility at XP (*n* = 3). (**E**) RNA-seq analysis shows FHT-1015-induced downregulation of HBV transcripts (*n* = 3). Statistical analyses were performed using one-way ANOVA with Tukey’s *post hoc* test. Significance levels: *P <.05, **P <.01, ***P <.001, ****P <.0001.

To determine whether the BAF complex directly engages the HBV minichromosome, we performed ChIP and DNA pull-down assays. ChIP-qPCR analysis in HBV infected HepG2-NTCP cells showed that SMARCC2 significantly accumulated in the CP, XP as well as SP2 but not SP1 of cccDNA, indicating the binding of SMARCC2 at these regions (Fig. 6A). Further ChIP-seq analysis in prcccDNA, pCre and pSMARCC2 transfected Huh7 cells with anti-SMARCC2 antibodies revealed significant enrichment of SMARCC2 at the EnhⅠ/XP (1200–1250 bp) and CP/EnhII (1700–1850 bp) regions of cccDNA, but not SP1 and SP2 (Supplementary Fig. S7A). DNA pull-down assays with biotinylated probes confirmed the specificity: EnhⅠ/XP and CP/EnhII DNA robustly captured SMARCC2, whereas SP1, SP2, or GFP control DNA failed to bind (Fig. 6B). Additionally, the positive control CMV-TATA box DNA, successfully pulled down SMARCC2 (Fig. 6B). Using biotinylated HBV probes matched with ChIP-seq peaks for DNA pull-down analysis, we found that truncated EnhⅠ/XP (1200–1250bp) and CP/EnhII (1700–1850 bp) regions can still interacted with SMARCC2 (Supplementary Fig. S7B). These findings establish EnhⅠ/XP and CP/EnhII as direct BAF anchoring sites on the viral minichromosome.

To assess BAF complex’s role in modulating cccDNA chromatin architecture, we performed assay for transposase-accessible chromatin with high throughput sequencing (ATAC-seq) in rcccDNA model cells (Fig. 6C) and HBV-infected PHHs (Fig. 6D). SMARCC2 overexpression in prcccDNA cells increased the chromatin accessibility specifically at the CP/EnhII region (positions 1606–1660 bp, 1.1-fold ↑, *P* <.01), consistent with its transcriptional activation (Fig. 6C). Conversely, SMARCA2/4 inhibition with FHT-1015 (2 μM) in PHHs infected with HBV (1000 VGE/cell) reduced the accessibility at CP/EnhII (positions 1459–1638 bp, 18%↓, *P* <.05) and SP1/SP2 (positions 2562–2762 bp, 10%↓, *P* <.001), but paradoxically increased the accessibility at EnhⅠ/XP (positions 700–900 bp, 1.3-fold↑, *P* < .0001) (Fig. 6D). This suggests BAF complex differentially remodels distinct regulatory domains, with CP/EnhII being its dominant site of action. Consistent with these epigenetic modifications, RNA-sequencing (RNA-seq) analysis revealed that FHT-1015 treatment elicited a substantial downregulation of global HBV transcripts (Fig. 6E).

### The BAF complex maintains transcriptional competence of HBV cccDNA via nucleosome remodeling

**Figure 7.**
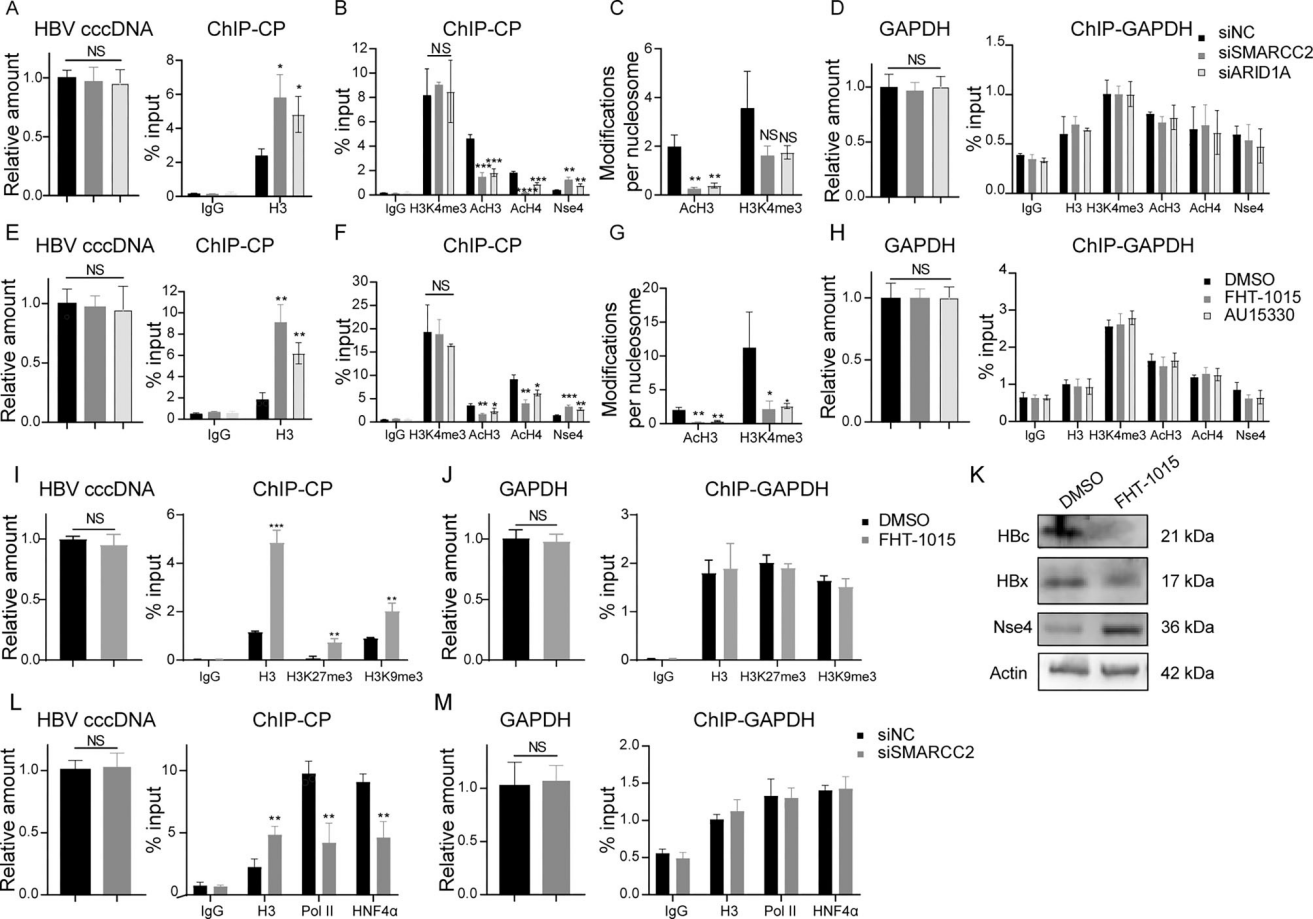
BAF regulates nucleosome organization and transcriptional machinery recruitment on HBV cccDNA. (**A–D**) Huh7-NTCP cells were transfected with siRNAs targeting SMARCC2 or ARID1A (cBAF subunits), followed by HBV infection (1000 VGE/cell). At 5 dpi: (**A**) cccDNA copy number (qPCR) and histone H3 occupancy (ChIP-qPCR). (**B**) Epigenetic marks (H3K4me3, AcH3, AcH4) and SMC5/6 complex (Nse4) binding to cccDNA. (**C**) Histone modification density normalized to nucleosome occupancy (AcH3/H3 and H3K4me3/H3 ratios). (**D**) Control locus (GAPDH) analysis: genomic DNA levels, histone H3 occupancy, epigenetic modifications, and SMC5/6 binding. (**E–H**) HBV-infected Huh7-NTCP cells (1000 vge) were treated with cBAF inhibitors (FHT-1015 or AU15330) and analyzed at 5 dpi: (**E–H**) Parallel assays as in panels (A)–(D), demonstrating compound-induced effects on cccDNA and GAPDH chromatin states. (**I–K**) HBV-infected HepG2-NTCP cells (1000 VGE/cell) were treated with FHT-1015 and analyzed at 5 dpi: (**I**) cccDNA copy number (qPCR), histone H3 occupancy and repressive histone markers (H3K27me3, H3K9me3, ChIP-qPCR). (**J**) Control locus (GAPDH) analysis: genomic DNA levels, histone H3 occupancy, repressive histone markers. (**K**) Viral protein (HBc and HBx) and Nse4 expression were assessed by WB. (**L–M**) Huh7-NTCP cells were transfected with siRNAs targeting SMARCC2, followed by HBV infection (1000 VGE/cell). At 5 dpi: (**L**) RNA polymerase II (Pol II) and hepatocyte nuclear factor 4α (HNF4α) enrichment on cccDNA. (**M**) Corresponding GAPDH locus controls. Data represent mean ± SD from three independent biological replicates. Statistical analyses were performed using two-tailed Student’s *t*-test (for two-group comparisons) or one-way ANOVA with Tukey’s *post hoc* test (for multiple comparisons). Significance levels: *P <.05, **P <.01, ***P <.001, ****P <.0001. The results from one representative experiment are shown. Experiments were repeated at least three times.

To dissect BAF complex’s role in HBV minichromosome dynamics, we analyzed nucleosomal occupancy and epigenetic modifications on cccDNA following SMARCC2 or ARID1A knockdown. ChIP revealed a two-fold increase in H3 density (*P* <.05) at CP of cccDNA, but not at SP1, SP2, or XP, indicating BAF complex specifically prevents nucleosome retention at CP (Fig. 7A and Supplementary Fig. S9A–G). Paradoxically, despite elevated nucleosome density, acetylated H3 (AcH3) and H4 (AcH4) marks at CP decreased by 50% (*P* <.01), while total H3K4me3 levels remained unchanged (Fig. 7B). Normalizing histone modifications to nucleosome density revealed reductions in AcH3 and H3K4me3 per nucleosome (Fig. 7C), suggesting BAF licenses both nucleosome eviction and epigenetic activation. Control experiments confirmed unaltered histone density and modifications at the glyceraldehyde-3-phosphate dehydrogenase (GAPDH) promoter [39] (Fig. 7D), underscoring cccDNA-specific regulation. As positive control, BAF depletion demonstrated increased histone density and decreased active modifications (H3K4me3 and AcH4) at the pS2 gene promoter of estrogen receptor 1 (ESR1) [40], a known BAF binding site (Supplementary Fig. S9H).

Given the structural maintenance of chromosomes 5/6 (SMC5/6) complex’s role in cccDNA repression [41, 42], we assessed its recruitment under BAF depletion. SMARCC2 or ARID1A knockdown enhanced Nse4 (SMC5/6 subunit) binding to cccDNA by 2-fold (*P* <.01; Fig. 7B), linking BAF loss to SMC5/6-dependent silencing. Pharmacological inhibition of SMARCA2/4 (FHT-1015) or PROTAC-mediated SMARCA2/4 degradation (AU15330) recapitulated these phenotypes: increased H3 density (3–5-fold ↑, *P* <.01), reduced AcH3/AcH4 (40%–50% ↓), enhanced Nse4 and Smc6 binding (Fig. 7E–G and Supplementary Fig. S9J). Meanwhile, repressive histone marks H3K27me3 and H3K9me3 on cccDNA were significantly upregulated (4–5-fold increase, *P* <.01) in FHT-1015-treated cells (Fig. 7I). Concurrently, the suppression of viral proteins (HBc and HBx) were accompanied by a restoration of Nse4 and Smc6 protein levels (Fig. 7K and Supplementary Fig. S9I). These results position BAF as a counterbalance to SMC5/6, maintaining transcriptional permissiveness. Control experiments confirmed unaltered histone modifications at the GAPDH promoter [39] (Fig. 7H and J), underscoring cccDNA-specific regulation of the compounds.

Consistent with chromatin compaction limiting transcriptional access, SMARCC2 knockdown reduced RNA polymerase II (Pol II) occupancy at cccDNA by 57 ± 11% (*P* <.01) and hepatocyte nuclear factor 4 alpha (HNF4α, a key HBV transcription factor) by 49 ± 9% (*P* <.01), while GAPDH promoter binding remained unaffected (Fig. 7L–M). This establishes BAF’s role in sustaining a transcriptionally active cccDNA architecture.

### Pharmacological inhibition of BAF complex suppresses HBV infection in liver-humanized mice

**Figure 8.**
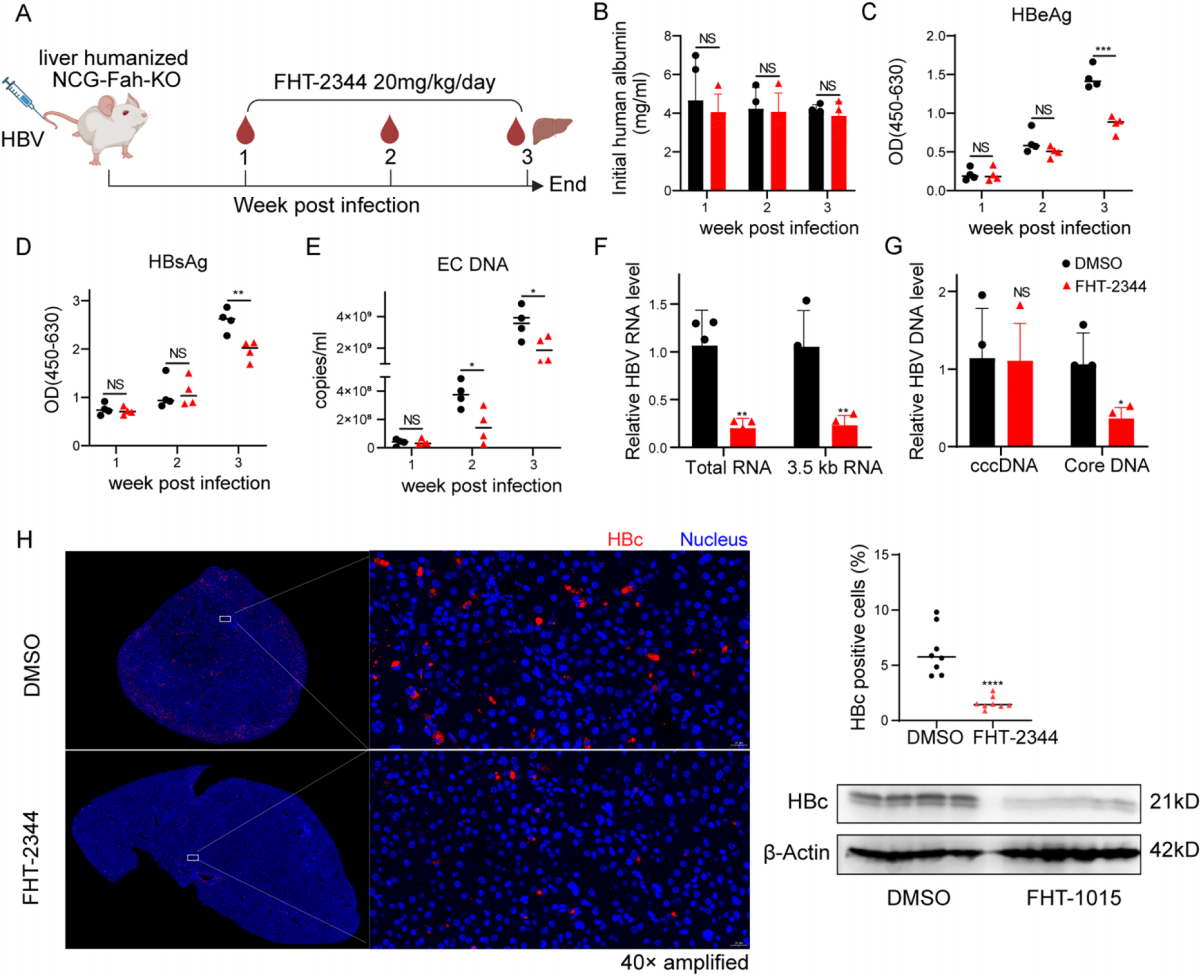
Pharmacological inhibition of BAF chromatin remodeling complex by FHT-2344 suppresses HBV infection in liver-humanized mice. (**A**) Experimental timeline for HBV challenge and FHT-2344 treatment in NCG-Fah-KO liver-humanized chimeric mice. Mice were inoculated with HBV (5 × 10^9^ VGE) and treated daily with FHT-2344 (20 mg/kg, oral gavage) or vehicle control (DMSO) from 7 to 21 days post-infection (dpi). (**B**) Longitudinal monitoring of human albumin levels in serum (DMSO: *n* = 4; FHT-2344: *n* = 4) by ELISA, demonstrating preserved liver engraftment during treatment. (**C–E**) Virological parameters in serum: (**C**) HBeAg, (**D**) HBsAg, and (**E**) extracellular HBV DNA (EC DNA) quantified by ELISA and qPCR, respectively. (**F–H**) Intrahepatic HBV replication intermediates at 5 dpi: (**F**) Total HBV RNA and 3.5 kb RNAs levels, and (**G**) cccDNA and core-associated DNA (Core DNA) quantified by qRT-PCR and qPCR. (**H**) Immunofluorescence and Western blot analysis of HBc protein expression in liver sections. Data represent mean ± SD from four independent biological replicates. Statistical significance was determined by two-tailed Student’s *t*-test (*P <.05, **P <.01, ***P <.001, ****P <.0001).

To evaluate the antiviral function of BAF inhibition, we tested the preclinical-stage SMARCA2/4 ATPase inhibitor FHT-2344—a pharmacokinetic-optimized analog of FHT-1015—in liver-humanized NCG-Fah-KO female mice (*n* = 4/group) engrafted with 100 000 human hepatocytes [43]. Mice inoculated with HBV (5 × 10^9^ VGE) received daily FHT-2344 (20 mg/kg, intragastric) for 14 days (Fig. 8A). Stable hepatic engraftment was confirmed via human serum albumin levels (Fig. 8B). Interim analysis at day 7 of treatment revealed FHT-2344 administration induced a 40% reduction in serum HBV DNA without significant modulation of HBsAg or HBeAg levels. By treatment day 14, marked virological suppression was observed with FHT-2344, evidenced by significant reductions in serum HBV markers including HBeAg (50% reduction, *P* <.001; Fig. 8C), HBsAg levels (35% reduction, *P* <.01; Fig. 8D), accompanied by a decrease in circulating HBV DNA (Fig. 8E). Hepatic analysis revealed concordant declines in intrahepatic HBV total RNA (70% reduction, *P* <.01), 3.5 kb RNAs (70% reduction, *P* <.01) and core associated DNA (60% reduction, *P* <.05), while cccDNA levels remained unaffected (Fig. 8F and G). HBcAg expression was similarly attenuated (Fig. 8H) compared to controls, indicating BAF inhibition selectively disrupts viral transcription and replication without eradicating the cccDNA reservoir.

## Discussion

The chromatin architecture of HBV cccDNA recapitulates eukaryotic transcriptional regulation, featuring NDRs at enhancer-promoter loci (EnhⅠ/XP, CP/EnhII) flanked by phased nucleosome arrays [7]. These NDRs, analogous to the −1/+1 nucleosome organization at cellular TSS, are essential for transcriptional initiation [44]. However, the mechanisms sustaining cccDNA NDR plasticity remained unresolved. Here, we identify SMARCC2—a scaffolding subunit of the ATP-dependent chromatin remodeling BAF complex—as an important regulator of HBV transcription. BAF complex directly binds CP/EnhII and EnhⅠ/XP, where it maintains the nucleosome depletion status, increases the accessibility and recruits the RNA Pol II. Genetic or pharmacological disruption of BAF complex compacts cccDNA chromatin, reduces histone acetylation, enables SMC5/6-mediated silencing and increases repressive histone markers, suppressing viral transcription across *in vitro* and *in vivo* models.

The BAF complex governs cccDNA transcriptional plasticity through ATP-dependent chromatin remodeling that dynamically balances nucleosome positioning and epigenetic activation. Our data reveal that SMARCC2, as a scaffolding subunit [45], anchors BAF complex to EnhⅠ/XP and Cp/EnhII regions, where it actively evicts nucleosomes to maintain NDRs—a process requiring its ATPase activity (Figs 6C and D, and 7A–H) [46]. This activity prevents nucleosome deposition over viral promoters, which would otherwise obstruct RNA Pol II and transcription factor (e.g. HNF4α) binding (Fig. 7L) [47]. Intriguingly, BAF’s nucleosome remodeling is coupled to histone acetylation and methylation: SMARCC2 depletion or BAF inhibition reduced AcH3 and AcH4 levels while enhanced H3K27me3 and H3K9me3 at CP (Fig. 7), suggesting BAF recruits or stabilizes HATs to preserve an active chromatin state [47]. This dual function—mechanical nucleosome displacement and epigenetic mark deposition—positions BAF as a master orchestrator of cccDNA’s transcriptional niche. Our promoter-reporter analyses indicated cBAF-specific regulation, yet the composition of BAF complexes on the native cccDNA minichromosome is likely more complex and may involve others. Therefore, comprehensive profiling is required to define the precise composition of BAF complexes recruited to cccDNA. Further investigation is also needed to elucidate the recruitment mechanisms and their relationship with clinical mutants, especially within the CP region.

The BAF complex is crucial in establishing NDRs required transcription activation. However, several reports also indicate its involvement in transcriptional repression [48–51]. Previous studies indicated that PRMT5 catalyzes symmetric dimethylation of histone H4 arginine 3 (H4R3me2s), regulating BAF complex binding to cccDNA [14]. This regulation potentially contributes to the PRMT5-mediated epigenetic suppression of cccDNA transcription. Given that transcription regulation is a multiple step process involving coordinated actions of numerous proteins, further investigation is required to reveal whether different types of histone epigenetic modifications mediate BAF complex recruitment, and whether this recruitment ultimately results in transcriptional activation or repression.

Critically, the BAF complex antagonizes the SMC5/6 complex, which restricts cccDNA transcription (Fig. 7B and F). SMARCC2 knockdown decreases the HBx level (Fig. 2E and Supplementary Fig. S3E), thereby alleviating HBx-mediated degradation of SMC5/6 complex. Indeed, the reduced HBc and HBx level in FHT-1015 treated cells were accompanied by a restoration of Nse4 and Smc6 protein levels (Fig. 7K and Supplementary Fig. S9I). Although the specific binding site(s) and recognition mechanism of SMC5/6 for cccDNA are not yet clear, this complex inhibit viral transcription, potentially by regulating histone modifications such as H3K27ac [52]. These observations suggest that epigenetic modifications on cccDNA likely influence the binding of both BAF and SMC5/6. Consistent with this model, inhibition of the BAF complex enhances SMC5/6 recruitment to cccDNA, promoting transcriptional silencing [41]. This antagonism mirrors the “see-saw” regulation observed in cellular stress responses, where chromatin remodelers and structural maintenance proteins dynamically compete for genomic loci [53]. BAF’s dominance under basal conditions ensures cccDNA remains transcriptionally active, while its inhibition tilts the balance toward SMC5/6-mediated repression. Such plasticity underscores cccDNA’s mimicry of host chromatin dynamics, exploiting endogenous regulatory conflicts to sustain persistence.

Notably, BAF’s regulatory influence extends beyond viral chromatin. SMARCC2 preferentially occupies promoter regions of host metabolic genes (Supplementary Fig. S7C and D) [43, 54, 55], with pathway analysis revealing enrichment of metabolism pathways, which may have potential impact on HBV replication (Supplementary Fig. S7D). Pharmacological BAF inhibition (FHT-1015) suppressed chromatin accessibility at 34 917 genomic loci [56, 57] and altered expression of 3694 host genes (Supplementary Fig. S8A–D). Integrated ATAC-/RNA-seq analysis identified 364 genes with coupled chromatin compaction and transcriptional suppression (Supplementary Fig. S8E), including downregulation of nine HBV-promoter-binding transcription factors, (e.g. HNF4α, peroxisome proliferator activated receptor gamma [PPARG]; Supplementary Fig. S8F) [58–63], suggesting that transcription factors such as HNF4α likely contributes to the suppression of viral transcription as an auxiliary mechanism to the direct chromatin remodeling of cccDNA. BAF’s dual role in sustaining viral transcription and host metabolic networks was complemented by BAF-mediated modulation of HBV-associated pathogenesis markers (Supplementary Table S3): (i) ISG15, whose serum levels correlate with cirrhosis progression and viral load [64]; (ii) the HBx-PAK1 metastatic axis [65]; and (iii) oncogenic drivers (TCF3, c-Jun, DOK1) of hepatocarcinogenesis [66–68]. These findings establish BAF inhibition as a promising therapeutic strategy for concurrently targeting HBV replication and its associated oncogenic pathways. Nonetheless, the potential for side effects must be carefully considered, given the vast number of genes regulated by the BAF complex—exemplified by HNF4α, a core transcription factor vital for liver differentiation and function. Additionally, investigating how BAF subunit expression correlates with viral load across different stages of CHB could prove highly useful for determining the optimal treatment window.

The observed differences in SMARCC2’s regulatory effects across experimental systems reflect model limitations and HBV’s chromatin context-dependency. In plasmid systems lacking viral chromatin, SMARCC2 selectively modulated CP and XP activity, whereas HBV recombinant cccDNA models revealed CP-centric regulation due to structural divergence from infection-formed cccDNA. In HBV-infected PHHs [69], FHT-1015 reduced CP, SP1 and SP2 accessibility but increased XP openness (Fig. 6D). Despite localized XP activation, global HBV transcription declined due to FHT-1015-induced suppression of CP and SP-dependent transcription factors (Supplementary Fig. S8F). XP accessibility may reflect viral counter regulation to amplify HBx [70, 71], though insufficient to rescue replication amid collapsed host metabolic support. The efficacy of BAF inhibitors (FHT-1015, FHT-2344) across HBV genotypes and models—including humanized mice—validates chromatin remodeling as a therapeutic vulnerability. Treatment with FHT-2344—a preclinical-stage SMARCA2/4 inhibitor in liver-humanized mice reduced serum HBV DNA (50%), HBeAg (50%), and HBsAg (35%) without eliminating cccDNA. This multilayered suppression, targeting both viral chromatin and host dependencies, overcomes limitations of current nucleos(t)ide analogs (3TC, ETV, TDF, TAF, TMF) [72]. Furthermore, FHT-1015 inhibits transcription from integrated HBV sequences in HepG2.2.15, Hep3B, and PLC/PRF/5 cells. Previous studies have shown that HBV integration sites are random [73] and fragments are often truncated, lacking viral regulatory elements [74–76]. Therefore, the observed reduction in HBsAg likely reflects BAF’ s broad role in regulating host chromatin architecture and transcription, which may indirectly influence the expression of nearby host-driven integrated HBV fragments, rather than a direct, specific mechanism targeting HBV integration. Given these genomic characteristics, the role of the BAF complex in regulating integrated HBV warrants further investigation in patient-derived samples. Persistent cccDNA retention highlights the need for combinatorial strategies, where BAF inhibitors may synergize with immune modulators (PEG IFNα) [77] or capsid blockers (GLS4) [78] to achieve functional cure.

In conclusion, our study establishes BAF as a master regulator of HBV persistence, licensing cccDNA transcription through direct chromatin remodeling and indirect metabolic reprogramming. By maintaining NDR accessibility, evicting repressive nucleosomes, and counteracting SMC5/6 silencing, BAF sustains a transcriptionally active viral minichromosome. Pharmacological inhibition disrupts this equilibrium, achieving epigenetic silencing of cccDNA while crippling host pathways essential for viral replication. The efficacy of BAF-targeted agents across preclinical models validates chromatin remodeling as a therapeutic axis and positions BAF as a first-in-class epigenetic target for HBV functional cure. Future work should explore combinatorial regimens to eradicate cccDNA and prevent viral rebound, advancing toward durable remission.

## Supplementary Material

gkag073_Supplemental_File

## Data Availability

Further information and requests for resources and reagents should be directed to and will be fulfilled by Xinwen Chen (chen_xinwen@gzlab.ac.cn). All raw and processed sequencing data generated in this study have been submitted to the Genome Sequence Archive in National Genomics Data Center, China National Center for Bioinformation/Beijing Institute of Genomics, Chinese Academy of Sciences (GSA; https://ngdc.cncb.ac.cn/bioproject/browse/PRJCA039069), which are publicly accessible at under accession number PRJCA039069.
