# Supplementary material for: Chromatin remodeler BAF maintains HBV cccDNA transcriptional competence and represents a therapeutic target
Source: Nucleic Acids Res. 2026 Feb 10;54(4):gkag073. doi: 10.1093/nar/gkag073 (PMC12887538; doi:10.1093/nar/gkag073)
Supplement: gkag073_Supplemental_File [file gkag073_supplemental_file.docx]

**Supplemental Figures And Tables**

**Chromatin remodeler BAF maintains HBV cccDNA transcriptional competence and represents a therapeutic target**

Dan Huang, Yi Zheng, Enze Deng, Xinlei Ji, Yecheng Zhang, Hao Sun, Yingshan Chen, Yongxuan Yao, Yuan Zhou, Mingxia Zhang, Zhe Zhou, Yinghua Chen, Dan Su, Xiaoying Fan, Xinwen Chen, Rongjuan Pei

This file includes:

Figure S1-9

Table S1-3


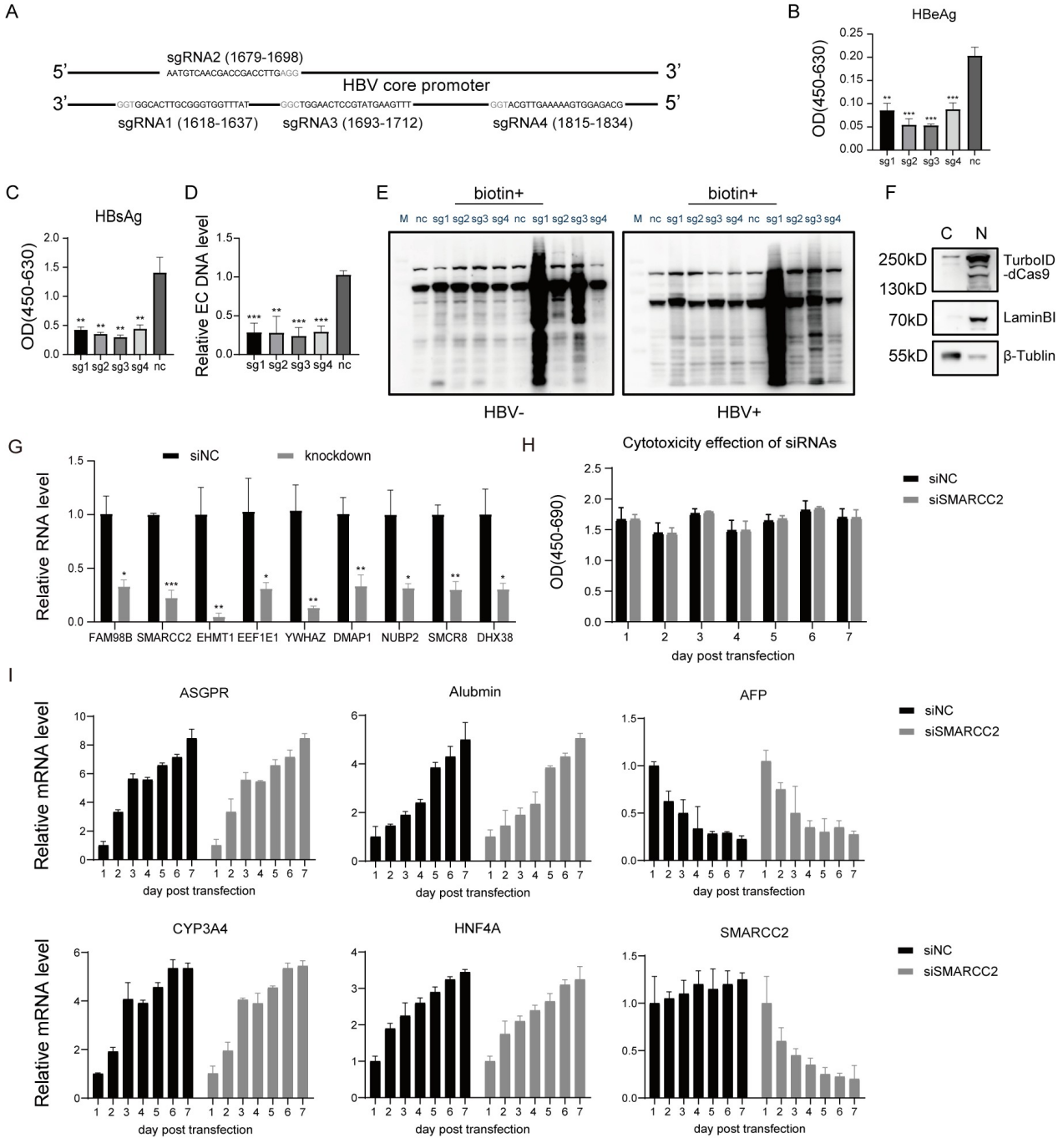


**Figure S1. Development of a CRISPR-based screening system to identify host factors regulating the HBV core promoter (CP).**

(A) sgRNA design. Four sgRNAs targeting the CP region were designed based on protospacer adjacent motif (PAM) sequences and synthesized for screening optimization.

(B-D) Functional validation of sgRNAs. HepAD38 cells were transfected with Cas9 plasmids encoding individual sgRNAs and analyzed at 48 h post-transfection: (B) Secreted HBeAg and (C) HBsAg levels (ELISA). (D) Extracellular HBV DNA (qPCR).

(E, F) System characterization. (E) Stable Huh7-NTCP cell lines expressing TurboID-dCas9 with individual sgRNAs were generated via lentiviral transduction. Cells infected with HBV (1000 VGE/cell), at 5 dpi. biotin-labeling proteins were analyzed by western blot, using streptavidin-HRP : sgRNA1 was selected for subsequent studies due to superior biotinylation efficiency upon HBV infection and biotin supplementation. (F) The correct nuclear localization of the fusion protein using anti-Flag antibody by western blot after nuclear cytoplasmic separation (C: cytoplasm; N: nucleus).

(G-I) siRNA knockdown efficiency, cytotoxicity analysis, and impact on liver cell differentiation. Huh7-NTCP cells were transfected with gene-specific siRNAs (or siNC control) for 2 days, then treated with DMSO for 5 days. (G) Knockdown efficiency of target genes (qRT-PCR) at 7 days post transfection. (H) Huh7-NTCP cells were transfected with siSMARCC2 or siNC control, WST-1 cell proliferation and cytotoxicity assay kit was used to detect cytotoxicity at the indicated time points. (I) Liver cell differentiation indicators and SMARCC2 mRNA level were detected via qRT-PCR with specific primers at indicated time points.

Data presentation: Mean ± SD from ≥ 3 independent biological replicates. Statistical significance was determined by two-tailed Student’s t-test (**p* < 0.05, ***p* < 0.01, ****p* < 0.001). All qRT-PCR data were normalized to Actin.


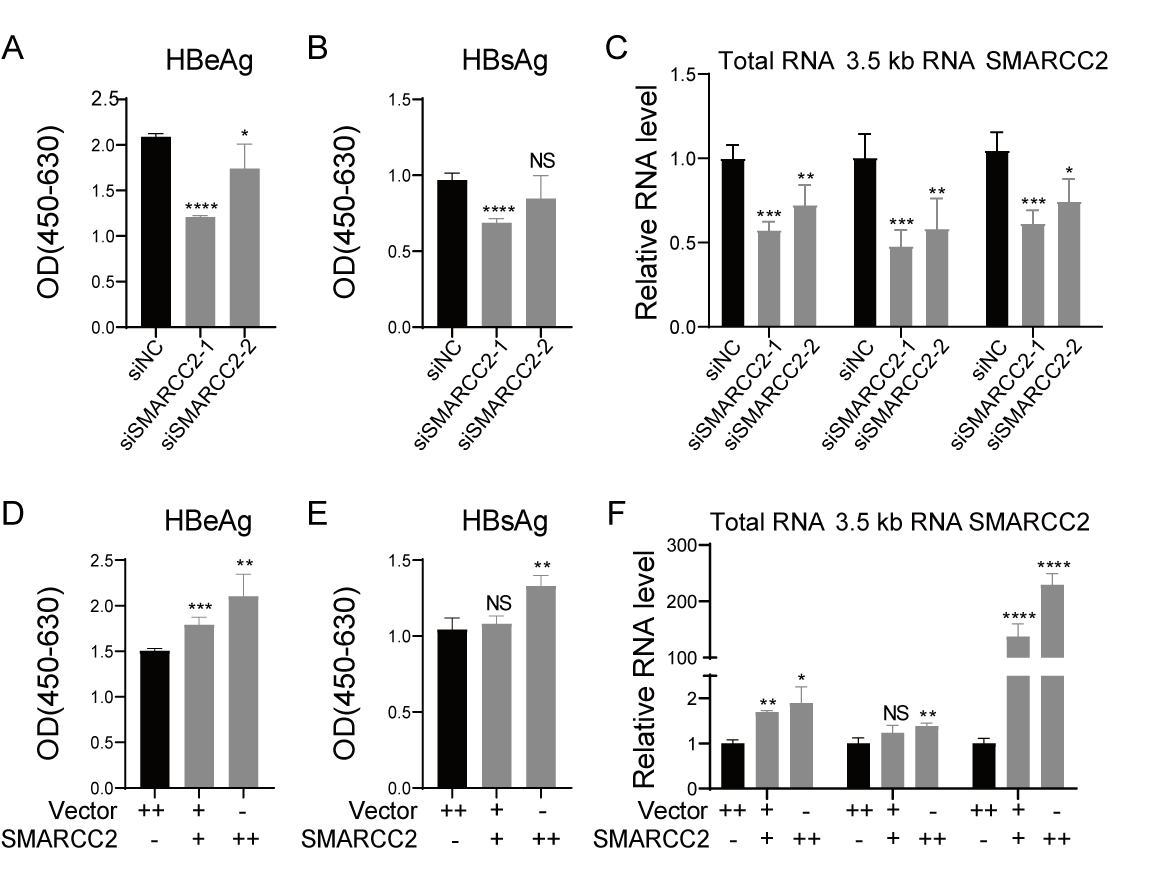


**Figure S2. SMARCC2 promotes HBV transcription in a Cre-mediated recombinant cccDNA system.**

(A-C) SMARCC2 knockdown attenuates HBV gene expression. Huh7 cells were co-transfected with prcccDNA (recombinant cccDNA precursor), pCre (Cre recombinase plasmid), and either siRNAs targeting SMARCC2 or negative control (siNC). Cells and supernatants were harvested at 48 h post-transfection（hpt）: (A) Secreted HBeAg and (B) HBsAg levels in supernatants (ELISA). (C) Intracellular HBV RNA (total RNA, 3.5 kb RNAs and SMARCC2 mRNA by qRT-PCR) and SMARCC2 knockdown efficiency.

(D-F) SMARCC2 overexpression enhances HBV transcription. Huh7 cells were co-transfected with prcccDNA, pCre, and either SMARCC2 overexpression plasmid (pSMARCC2) or empty vector. Cells and supernatants were harvested at 48 hpt: (D) Secreted HBeAg and (E) HBsAg levels (ELISA). (F) Intracellular HBV RNAs and SMARCC2 mRNA (qRT-PCR).

Data presentation: Mean ± SD from ≥3 independent biological replicates. Statistical one-way ANOVA with Tukey's test (**p* < 0.05, ***p* < 0.01, ****p* < 0.001, *****p* < 0.0001). All qRT-PCR data normalized to Actin. The results from one representative experiment are shown. Experiments were repeated at least three times.


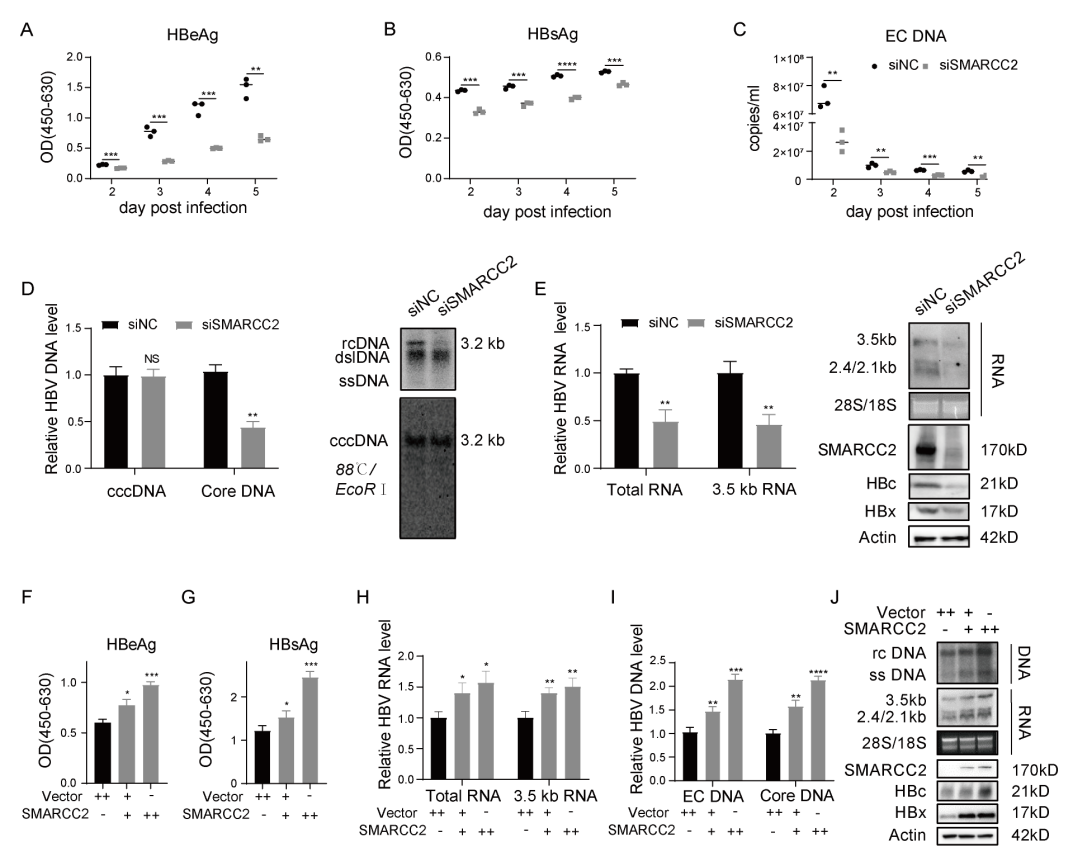


**Figure S3. SMARCC2 regulates HBV infection and replication in hepatoma cells.**

(A-E) SMARCC2 knockdown suppresses HBV infection in HepG2-NTCP cells. HepG2-NTCP cells were transfected with SMARCC2-targeting siRNA (siSMARCC2) or negative control (siNC) for 48 h, then infected with HBV (1000 VGE/cell) for 16 h. After PBS washing, cells were maintained in complete medium: (A) Daily HBeAg and (B) HBsAg secretion (ELISA). (C) Extracellular HBV DNA (qPCR). (D) Intracellular HBV DNA at 5 dpi: cccDNA and core-associated DNA (qPCR and Southern blot). (E) HBV RNA species: total RNA and 3.5 kb RNAs (qRT-PCR and Northern blot). Inset: SMARCC2 knockdown efficiency and viral protein (HBc/HBx) expression (Western blot).

(F-J) SMARCC2 overexpression enhances HBV replication. HepG2 cells were co-transfected with HBV replicon plasmid (pSM2, 1 μg) and either SMARCC2 expression plasmid (pSMARCC2, 1 μg) or empty vector: (F) HBeAg and (G) HBsAg secretion (ELISA). (H) HBV RNA: total RNA and 3.5 kb RNAs (qRT-PCR). (I) Viral DNA forms: extracellular and core-associated DNA (qPCR). (J) SMARCC2 overexpression and viral markers (Western/Northern/Southern blots).

Data presentation: Mean ± SD (n = 3 biological replicates). Statistics: two-tailed t-test (A-E) or one-way ANOVA with Tukey's test (F-I) (**p* < 0.05, ***p* < 0.01, ****p* < 0.001, *****p* < 0.0001). All qRT-PCR data were normalized to Actin. The results from one representative experiment are shown. Experiments were repeated at least three times.


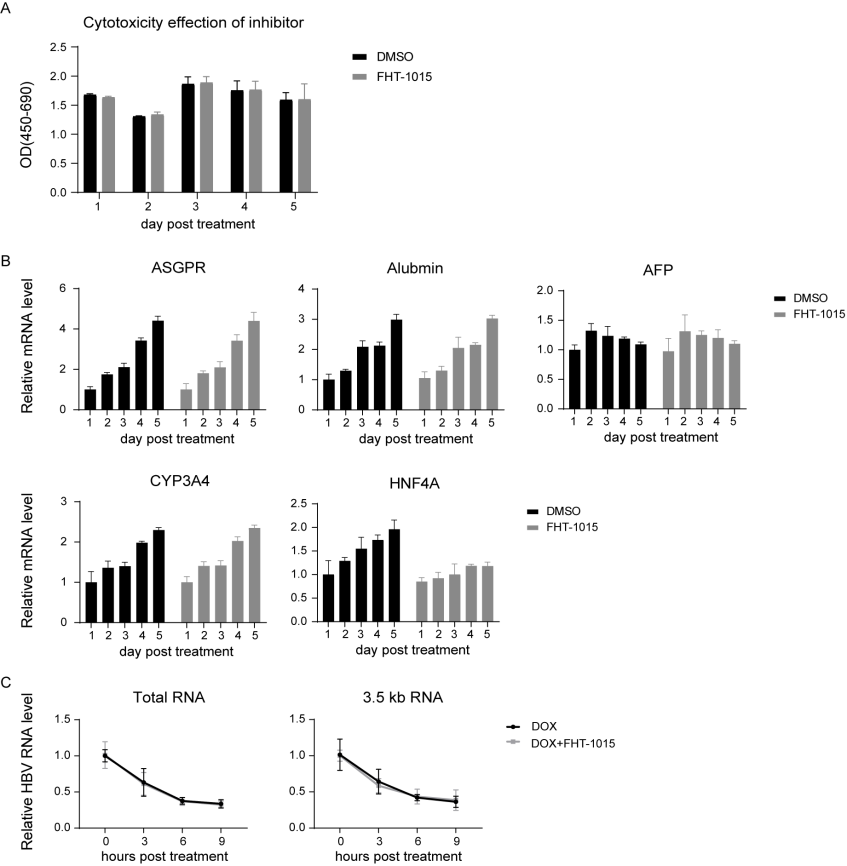


**Figure S4. The effect of FHT-1015 on cytotoxicity, liver cell differentiation and HBV RNA stability.**

1. B) Huh7-NTCP cells were treated with DMSO or FHT-1015 for 5 days, samples were collected daily to detect cytotoxicity and liver cell differentiation indicators. (A)WST-1 cell proliferation and cytotoxicity assay kit was used to detect cytotoxicity at the indicated time points. (B) Liver cell differentiation indicators mRNA level was detected via qPCR with specific primers at indicated time points.
2. After seeding for 48 hours, HepAD38 cells were treated with DOX or DOX and FHT-1015 for 9 hours. Cells were collected at indicated time points. The level of HBV total RNA and HBV 3.5 kb RNAs were measured by qRT-PCR with specific primers.

Data presentation: Mean ± SD (n = 3 biological replicates). All qRT-PCR data were normalized to Actin and compared with DMSO treated group normalized at 1 day post treatment.


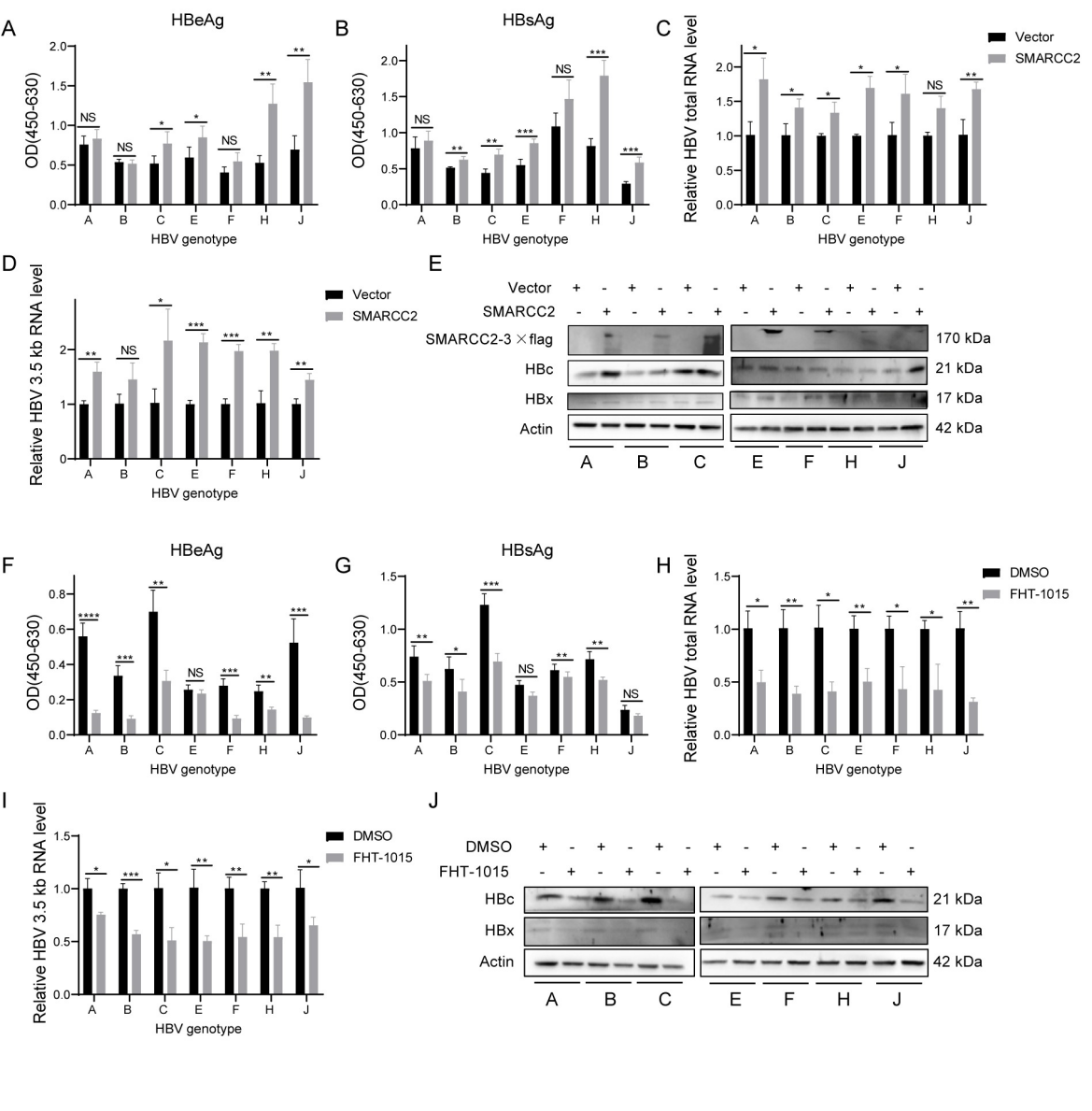


**Figure S5**. **The impact of BAF complex on HBV replication across multiple genotypes**.

Huh7 cells were co-transfected with HBV replicon plasmid (genotype A, B, C, E, F, H or J, 0.4 μg) together with SMARCC2 expression plasmid (pSMARCC2-3×flag, 0.4 μg) (A to E), or treated with FHT-1015 (F to J). At 48 hours post transfection: HBeAg (A, F) and HBsAg (B, G) secretion were quantified by ELISA. HBV RNA species (C-D, H-I) were detected by qRT-PCR. SMARCC2 and viral protein (HBc/HBx) expression level (E, J) were quantified by Western blot.

Data presentation: Mean ± SD (n = 3 biological replicates). All qRT-PCR data normalized to Actin and compared with control group.


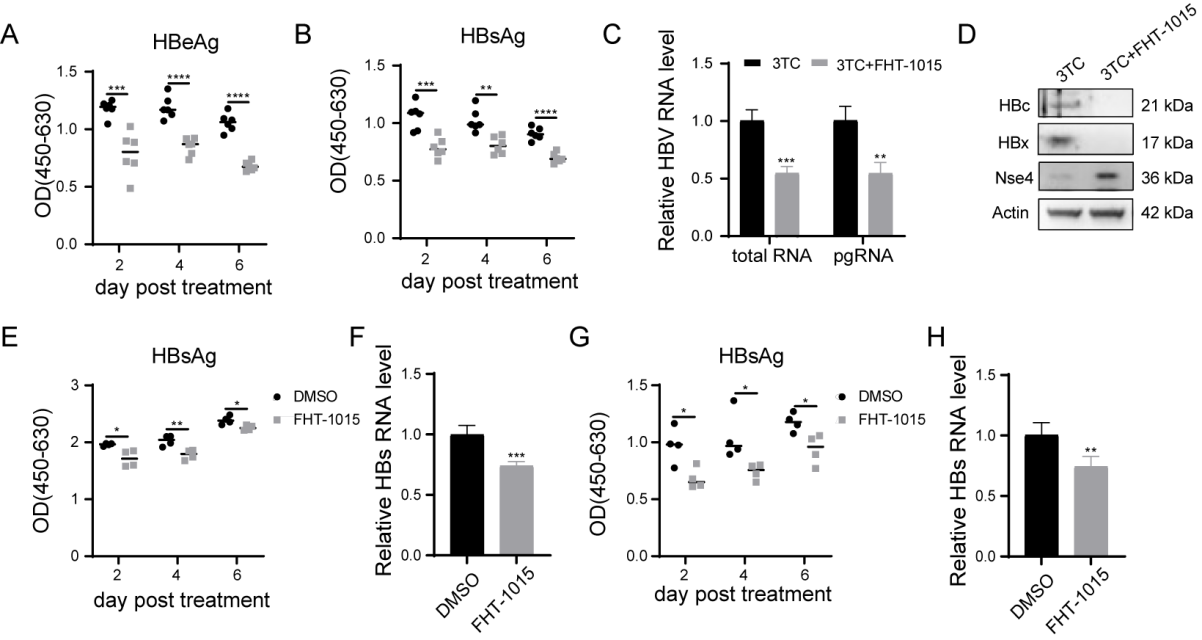


**Figure S6. The inhibitory effect of FHT-1015 on integrated HBV.**

(A-D) HepG2.2.15 cells were treated with 3TC (1 μM) and then treated with DMSO or FHT-1015 (2 μM). The supernatants were refreshed every two days. Cells were collected at 6 days post treatment. HBeAg (A) and HBsAg (B) levels in supernatants were quantified by ELISA. HBV RNA species (C) were detected by qRT-PCR. Nse4 and viral protein (HBc, HBx) expression levels (D) were determined by Western blot.

(E-F) Hep3B and (G-H) PLC/PRF/5 cells were treated with DMSO or FHT-1015 (2 μM). The supernatants were refreshed every two days. Cells were collected at 6 days post treatment. HBsAg (E, G) levels in supernatants were quantified by ELISA. HBs RNA (F, H) was detected by qRT-PCR.

Data presentation: Mean ± SD (n ≥ 3 biological replicates). All qRT-PCR data were normalized to Actin and compared with control group.


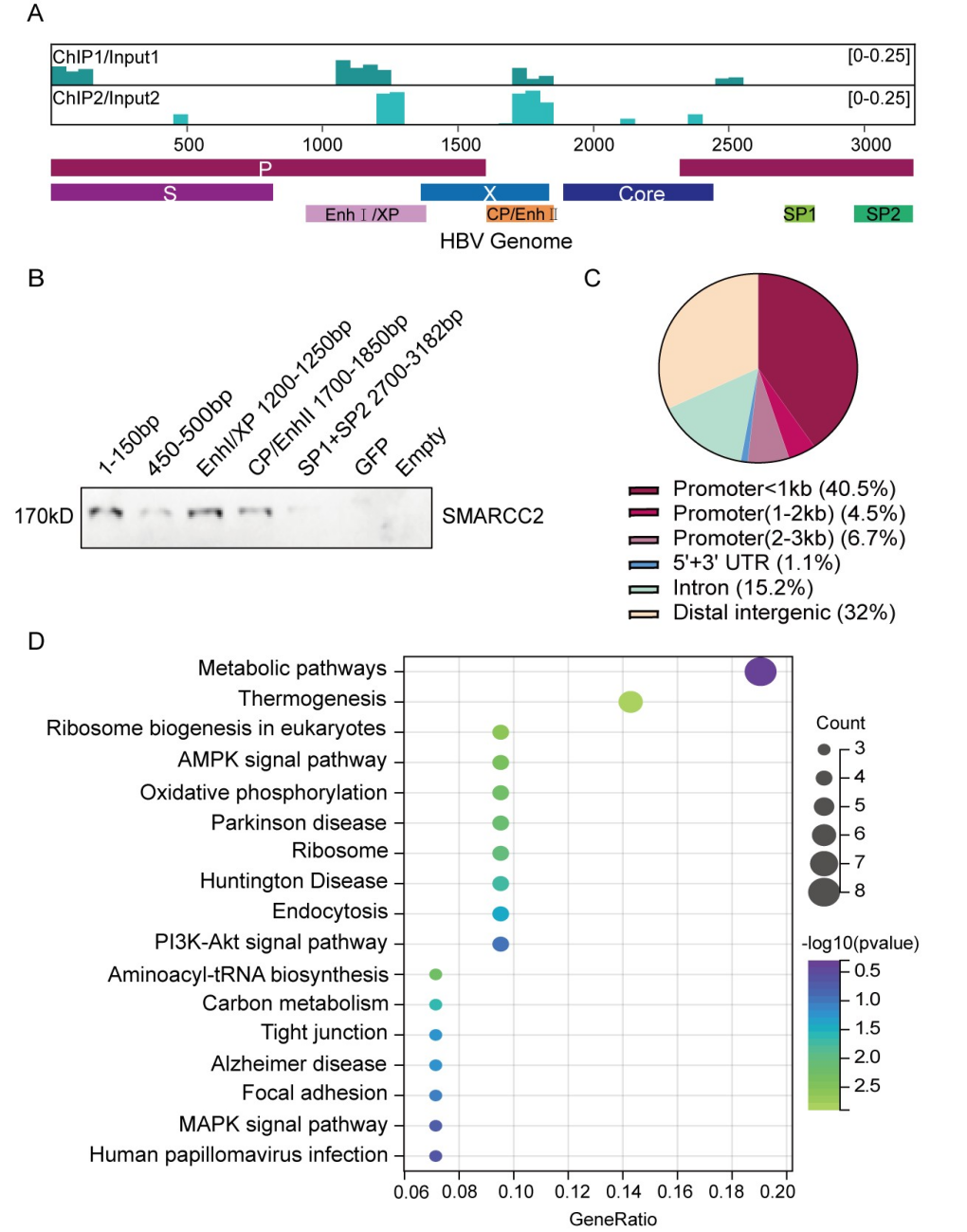


**Figure S7. Genome-wide profiling reveals SMARCC2 binding to HBV regulatory elements and cellular metabolic gene promoters.**

1. C) SMARCC2 chromatin occupancy analysis in HBV rcccDNA system. Huh7 cells were co-transfected with prcccDNA (recombinant cccDNA precursor), pCre (Cre recombinase plasmid) and pSMARCC2-3×Flag (SMARCC2 overexpression plasmid). ChIP-seq was performed at 48 hpt using anti-SMARCC2 antibody. (A) SMARCC2 binding landscape on HBV rcccDNA (2 biological replicates were shown). (B) DNA pulldown assay using biotinylated HBV probes matching ChIP-seq peaks (A). (C) Genomic distribution of SMARCC2 peaks in host chromatin, categorized by genomic features (promoters, enhancers, etc.). (D) Pathway enrichment analysis (KEGG) of SMARCC2-bound cellular genes, highlighting metabolic pathways. ChIP-seq peaks called using MACS2 (q < 0.05). KEGG analysis performed with clusterProfiler. All experiments included biological replicates (n = 2).


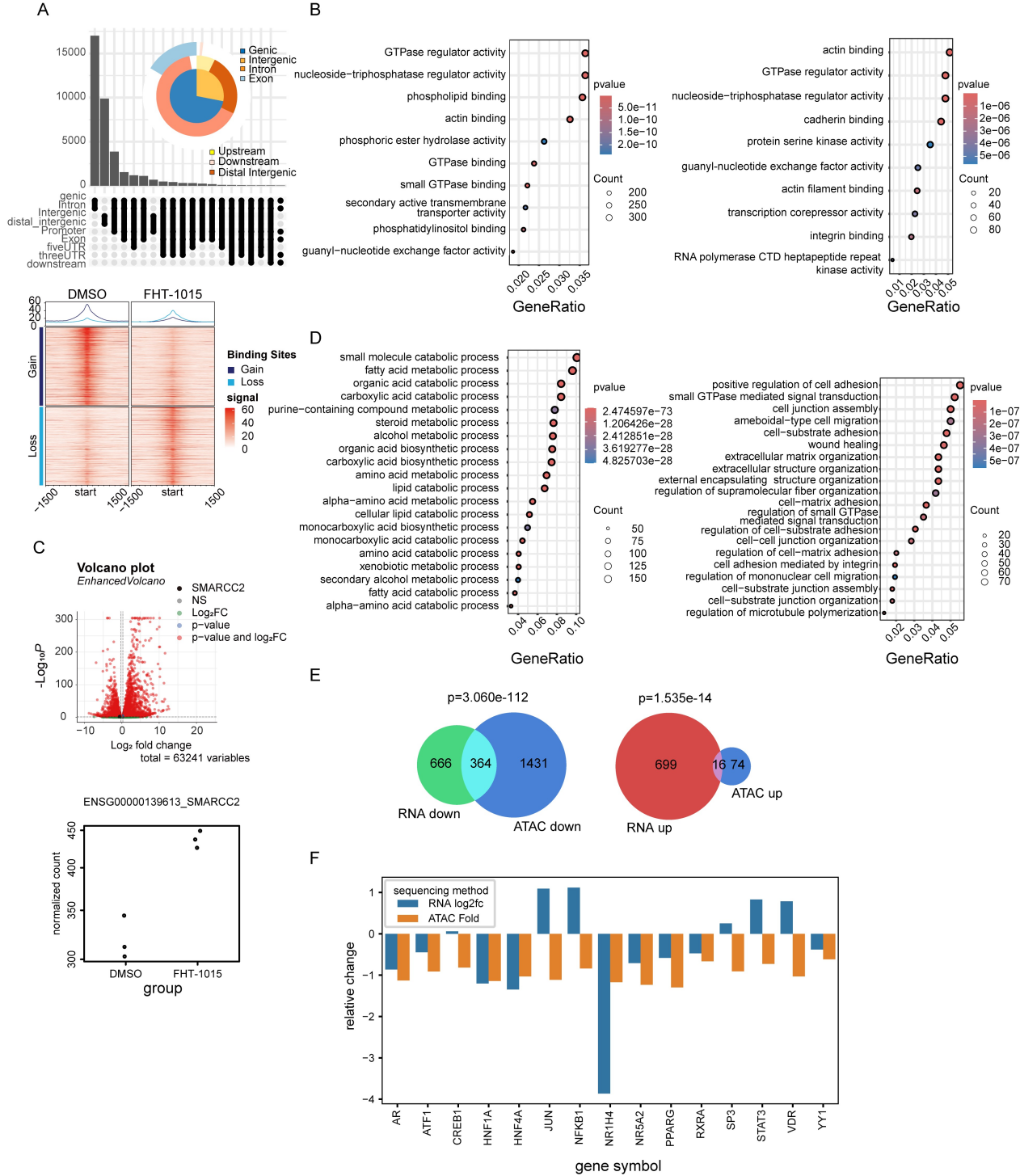


**Figure S8. Genome-wide profiling reveals FHT-1015-mediated alterations in chromatin accessibility and transcriptional regulation.**

(A-F) Multi-omics profiling of FHT-1015 treated cells. PHHs were infected with HBV (1000 VGE/cell, HepAD38-derived) for 12 days with DMSO or FHT-1015. At 12 days post infection (12 dpi.), PHHs were harvested for ATAC-seq and RNA-seq (n=3).

(A) Genomic distribution of chromatin accessibility changes between DMSO (control) and FHT-1015 treated groups.

(B) Functional enrichment of differentially accessible regions. Gene Ontology (GO) analysis of genes associated with FHT-1015-induced chromatin changes. Top significantly enriched biological processes shown.

(C) Transcriptional regulation of SMARCC2. Volcano plot demonstrating significant upregulation of SMARCC2 mRNA in FHT-1015 group. Dashed lines indicate significance thresholds (FDR < 0.05, log2FC = 0.4529).

(D) Pathway analysis of differentially expressed genes. GO enrichment of FHT-1015-responsive genes from RNA-seq data. Highlighted pathways related to viral transcription and chromatin remodeling (FDR < 0.05, log2FC > 1).

(E) Integrative analysis of chromatin accessibility and gene expression. Left: Genes with coordinated decrease in both mRNA expression and chromatin accessibility. Right: Genes with coordinated increase in both mRNA expression and chromatin accessibility.

(F) Regulation of HBV transcription factors. Upper: HBV-associated transcription factors showing increased expression/accessibility. Lower: Factors showing decreased expression/accessibility.

ATAC-seq and RNA-seq performed in biological triplicates. FDR < 0.05 considered statistically significant.


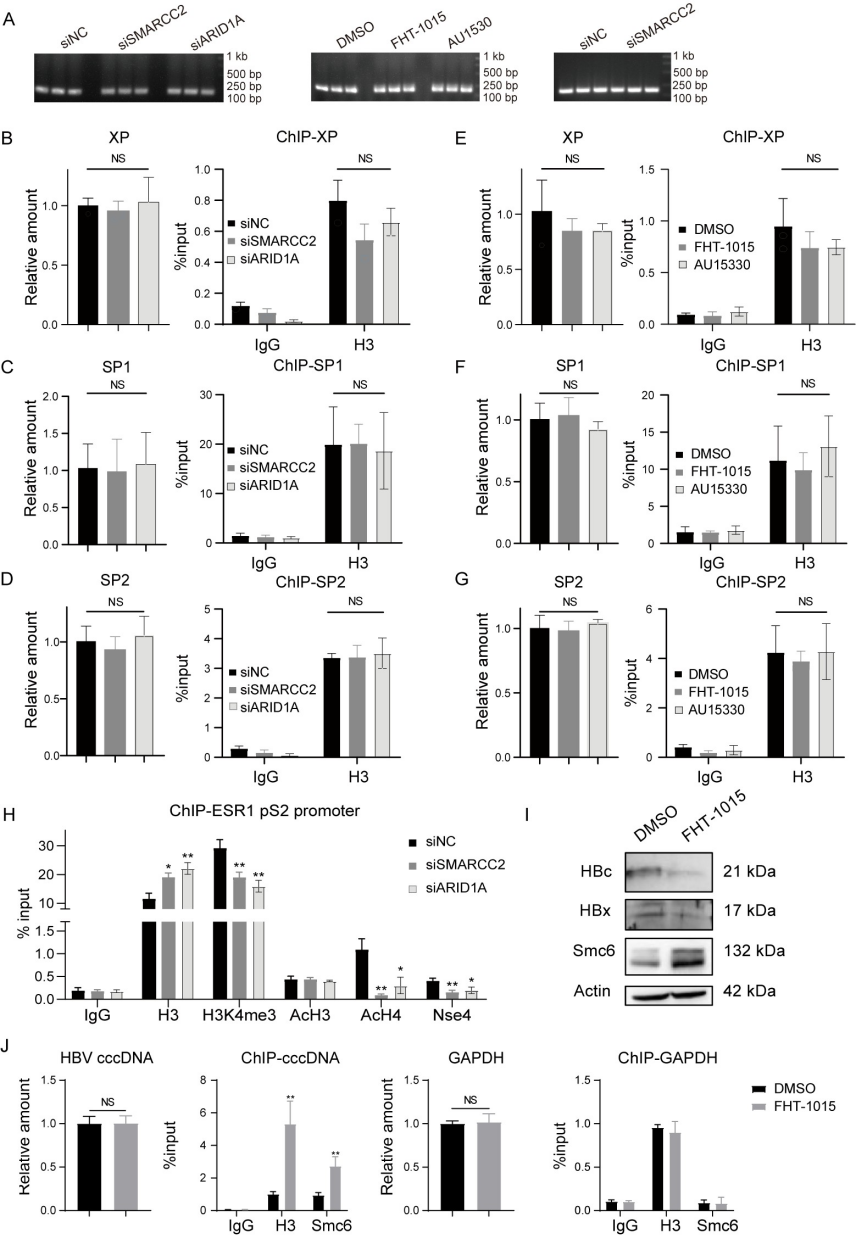


**Figure S9. Targeting cBAF complex does not alter nucleosome occupancy at HBV XP, SP1 and SP2 promoter but affect known binding sites.**

Chromatin digestion of ChIP experimental samples were identified by agarose gel electrophoresis (A). Huh7-NTCP cells were infected with HBV and then transfected with indicated siRNA (B-D), or treated with different compounds (E-G). At 5 days postinfection (dpi.), cross-linked chromatin was immunoprecipitated with anti-H3 antibody or IgG. The levels of histone H3 bound to cccDNA (B, E) XP, (C, F) SP1, (D, G) SP2, the levels of histone H3 and indicated epigenetic modification bound to ESR1 pS2 promoter (H) in different groups were determined via qPCR and ChIP-qPCR analysis with specific primers. (I) Viral protein (HBc and HBx) and Smc6 expression were assessed by Western blotting. (J) The levels of cccDNA, GAPDH, histone H3 and Smc6 bound to cccDNA and GAPDH control in different groups were determined via qPCR and ChIP-qPCR.

Data presentation: Mean ± SD (n = 3 biological replicates). Statistics: one-way ANOVA with Tukey's test (A-F) (**p* < 0.05, ***p* < 0.01, ****p* < 0.001, *****p* < 0.0001). The results from one representative experiment are shown. Experiments were repeated at least three times.

| **Table S1 Key resources** | | |
| --- | --- | --- |
| **REAGENT or RESOURCE** | **SOURCE** | **IDENTIFIER** |
| **Antibodies** |  |  |
| Rabbit anti-HBc | abcam | ab115992 |
| Rabbit anti-HBc | Dr. Yuchen Xia (Wuhan University) | N/A |
| Mouse anti-LaminBⅠ | Proteintech | 12987-1-AP |
| Mouse anti-β-tubulin | Cell Signal Technology | 2128s |
| Rabbit anti-HBx | Biovendor | RD981038100 |
| Rabbit anti-SMARCC2 | ABclonal | A1967 |
| Rabbit anti-SMARCC2 | CUSABIO | CSB-PA851527ESR2HU |
| Mouse anti-Flag | Proteintech | 66008-4-Ig |
| Mouse anti-β-actin | Proteintech | 66009-1-Ig |
| Rabbit anti-H3 | Cell Signal Technology | 4620 |
| H3K4me3 | Abcam | ab8580 |
| AcH3 | Millipore | 06-599 |
| AcH4 | Millipore | 06-598 |
| Nse4 | Abgent | AP9909A |
| Anti-SMC6 | Santa Cruz | sc-365742X |
| Rabbit IgG | Cell Signal Technology | 2729 |
| Rabbit anti-HNF4α | abcam | ab200142 |
| Rabbit anti-RNA polymerase II | abcam | ab5131 |
| Streptavidin-HRP concentration | ABclonal | RM17481 |
| **Cell lines** |  |  |
| Huh7 | Laboratory retention | N/A |
| HepG2 | AORUICELL | ORC1149 |
| Hep3B | AORUICELL | ORC0821 |
| PLC/PRF/5 | AORUICELL | ORC0269 |
| Huh7-NTCP | Laboratory retention | N/A |
| HepG2-NTCP | Dr. Yuchen Xia (Wuhan University) | N/A |
| HepAD38 | AORUICELL | ORC1159 |
| PHH | Liver Biotechnology | LV-PHH001 |
| **plasmids** |  |  |
| prcccDNA | Dr. Qiang Deng (Fudan University) | N/A |
| pCre | Dr. Qiang Deng (Fudan University) | N/A |
| pCMV-T7-MCS-3×FLAG-Neo | MiaoLing Plasmid Platform | P1303 |
| pCMV-SMARCC2(human)-3×FLAG-Neo | MiaoLing Plasmid Platform | P59382 |
| pCMV-SMARCA4(human)-3×FLAG-Neo | MiaoLing Plasmid Platform | P46487 |
| pCMV-SMARCA4(human)-K785R-3×FLAG-Neo | Self-made | N/A |
| pCP | Dr. Mengji Lu (University Hospital of Essen) | N/A |
| pXP | Dr. Mengji Lu (University Hospital of Essen) | N/A |
| pSP1 | Dr. Mengji Lu (University Hospital of Essen) | N/A |
| pSP2 | Dr. Mengji Lu (University Hospital of Essen) | N/A |
| pSM2 | Dr. Mengji Lu (University Hospital of Essen) | N/A |
| pHBV A | Dr. Yu Chen (Wuhan University) | N/A |
| pHBV B | Dr. Yu Chen (Wuhan University) | N/A |
| pHBV C | Dr. Yu Chen (Wuhan University) | N/A |
| pBlueBac-HBV E | Dr. Jieliang Chen (Fudan University) | N/A |
| pUC57 HBV F | Dr. Jieliang Chen (Fudan University) | N/A |
| pUC57 HBV H | Dr. Jieliang Chen (Fudan University) | N/A |
| pUC57 HBV J | Dr. Jieliang Chen (Fudan University) | N/A |
| **siRNAs** |  |  |
| siSMARCC2 | GGAUGAAUGAGGAAGACUA/dT//dT/ | UAGUCUUCCUCAUUCAUCC/dT//dT/ |
| siARID1A | CAAUGGAUCAGAUGGGCAA/dT//dT/ | UUGCCCAUCUGAUCCAUUG/dT//dT/ |
| siSMARCA4 | UAGCAUUGAGGGCUGUCUCCA/dT//dT/ | UGGAGACAGCCCUCAAUGCUA/dT//dT/ |
| siARID2 | CGUACCUGUCUUCGUUUCCUA/dT//dT/ | UAGGAAACGAAGACAGGUAGC/dT//dT/ |
| siBRD9 | ACUCCAGUUACUAUGAUGAC/dT//dT/ | GUCAUCAUAGUAACUGGAGU/dT//dT/ |
| **Primers for ChIP-qPCR** |  |  |
| GAPDH promorer | 5’-TACTAGCGGTTTTACGGGCG-3’ | 5’-TCGAACAGGAGGAGCAGAGAGCGA-3’ |
| ESR1 pS2 promoter | 5’-GGGGCACATAAGGCAGCACATT-3’ | 5’-GCTGGATAGAGGCTGAGTTTCACG-3’ |
| HBV CP | 5’-GTGCACTTCGCTTCACCTCT-3’ | 5’-AGCTTGGAGGCTTGAACAGT-3’ |
| HBV XP | 5’-TCATGGGCCATCAGCGCATG-3’ | 5’-TGGCAGCACAGCCTAGCAGC-3’ |
| HBV SP1 | 5’-GGCCCACTCACAGTTAATGAG-3’ | 5’-CCCAAGAATATGGTGACCCA-3’ |
| HBV SP2 | 5’-GGCCAGACGCCAACAAGGTA-3’ | 5’-TTCCACTGCATGGCCTGAGG-3’ |
| **Primers for qPCR (RT-PCR)** |  |  |
| Human β-actin | 5’-GAAATCGTGCGTGACATTAA-3’ | 5’-AAGGAAGGCTGGAAGAGTG-3’ |
| Human SMARCC2 | 5’-ACTGCCGATCAAATGTTTCCT-3’ | 5’-ACAGGCAATTATTCTGCACCAAG-3’ |
| HBV total RNA | 5’-CCGTCTGTGCCTTCTCATCTGC-3’ | 5’-ACCAATTTATGCCTACAGCCTCC-3’ |
| HBV pgRNA | 5’-CTGGGTGGGTGTTAATTTGG-3’ | 5’-TAAGCTGGAGGAGTGCGAAT-3’ |
| HBV rcDNA | 5’-ACCAATCGCCAGTCAGGAAG-3’ | 5’-ACCAGCAGGGAAATACAGGC-3’ |
| HBV cccDNA | 5’-CTCCCCGTCTGTGCCTTCT-3’ | 5’-GCCCCAAAGCCACCCAAG-3’ |
| Human ASGPR | 5’-TGCTGCTTGTGGTTGTCT-3’ | 5’-CTTCATCTTTCTTCCCACATT-3’ |
| Human Alubmin | 5’-ACTATCTATCCGTGGTCCTGA-3’ | 5’-TCTTGATTTGTCTCTCCTTCT-3’ |
| Human AFP | 5’-CCAACAGGAGGCCATGCTT-3’ | 5’-GAATGCAGGAGGGACATATGTTT-3’ |
| Human CYP3A4 | 5’-TCCATTCCTCATCCCAATTCTTGA-3’ | 5’-TCCACTCGGTGCTTTTGTGT-3’ |
| Human HNF4A | 5’-GGCCAAGTACATCCCAGCTT-3’ | 5’-TCATTGCCTAGGAGCAGCAC-3’ |
| **Sequence for DNA pull down** |  |  |
| CMV | IBIOdT/GACATTGATTATTGACTAGTTATTAATAGTAATCAATTACGGGGT | IBIOdT/AGCTCTGCTTATATAGACCTCCCACC |
| GFP | IBIOdT/ATGGTGTCTAAGGGCGAAGAGCTGTT | IBIOdT/TTACTTGTACAGCTCGTCCATGCCGAG |
| HBV EnhⅠ/XP | IBIOdT/GAATGTTTTAGAAAACTTCCTATTAACAGGCCT | IBIOdT/CTATCCCGCAAATATACATCGTTTCCATG |
| HBV CP/EnhⅡ | IBIOdT/GACCACCGTGAACGCCCA | IBIOdT/TTTCACCTCTGCCTAATCATCTCTTGT |
| HBV SP1 | IBIOdT/AACATCTAGTTAATCATTACTTCCAAACTAGACACTATTTACACACTCTATGGAAGGCGGGTATATTATATAAGAGAGAAACAACACATAG | IBIOdT/CTATGTGTTGTTTCTCTCTTATATAATATACCCGCCTTCCATAGAGTGTGTAAATAGTGTCTAGTTTGGAAGTAATGATTAACTAGATGTT |
| HBV SP2 | IBIOdT/GGCCAGACGCCAACAAGGTAGG | IBIOdT/TTCCACTGCATGGCCTGAGG |
| HBV 1-150 | IBIOdT/CTCCACAACCTTCCACCAAACTCT | IBIOdT/AGCGCAGGGTCCCCAATC |
| HBV 450-500 | IBIOdT/ACTATCAAGGTATGTTGCCCGTTTGTCCTCTAATTCCAGGATCCTCAACAA | IBIOdT/TTGTTGAGGATCCTGGAATTAGAGGACAAACGGGCAACATACCTTGATAGT |
| HBV EnhⅠ/XP 1200-1250 | IBIOdT/ACTGGCTGGGGCTTGGTCATGGGCCATCAGCGCATGCGTGGAACCTTTTC | IBIOdT/GAAAAGGTTCCACGCATGCGCTGATGGCCCATGACCAAGCCCCAGCCAGT |
| HBV CP/EnhⅡ 1700-1850 | IBIOdT/GGCATACTTCAAAGACTGTTTGTTTAAA | IBIOdT/TTTCACCTCTGCCTAATCATCTCTTGT |
| HBV SP1+SP2 | IBIOdT/AACATCTAGTTAATCATTACTTCCAAACTAGACAC | IBIOdT/TTCCACTGCATGGCCTGAGG |
| “IBIOdT” refers to the nucleotide-adding-biotin modification. | | |

| **Table S2 Significantly enriched proteins** | | |
| --- | --- | --- |
| **Gene** | **HBV3d+/HBV3d- Fc** | **P-value** |
| SHTN1 | 1.548988126 | 0.001102425 |
| DAD1 | 1.627520431 | 0.034348559 |
| BNIP3 | 1.923982311 | 0.035186901 |
| CIAPIN1 | 1.629770048 | 0.014380178 |
| TDP2 | 1.671932516 | 0.032734168 |
| TXN | 1.508282773 | 0.013503802 |
| TRIM25 | 1.752469164 | 0.003574051 |
| EMC1 | 1.907853149 | 0.001908784 |
| TNRC6A | 1.590332677 | 0.028615764 |
| PDZD8 | 3.484011364 | 0.0059899 |
| NDRG1 | 1.595351607 | 0.020877406 |
| DHX38 | 1.560857172 | 0.008261752 |
| MYO19 | 1.51298754 | 0.020838397 |
| EFHD1 | 1.752808567 | 0.009651776 |
| TMEM109 | 1.643984375 | 0.044339408 |
| ARFGAP3 | 1.541000936 | 0.001660131 |
| TXLNG | 1.732897001 | 0.047212486 |
| CDV3 | 1.617970243 | 0.005506903 |
| SNX12 | 1.904400488 | 0.019867864 |
| NUDC | 1.52656505 | 0.029463509 |
| SNX9 | 1.595569099 | 0.012656708 |
| CHCHD3 | 16.79005912 | 0.01959432 |
| NUBP2 | 4.507910183 | 0.012371681 |
| ELOVL2 | 2.017670867 | 0.00179005 |
| SRR | 2.095408597 | 0.03769718 |
| Cathepsin D | 2.704471056 | 0.012959542 |
| CDCA2 | 3.41878144 | 0.021366742 |
| LRSAM1 | 64.63427133 | 0.000462056 |
| SMCR8 | 85.6721169 | 9.84876E-06 |
| DMAP1 | 3.507712679 | 0.021051461 |
| BPNT2 | 6.187161421 | 0.004058321 |
| CRKL | 2.487757183 | 0.030257235 |
| PFDN5 | 2.800131502 | 0.001925636 |
| NSFL1C | 6.081771909 | 0.010610983 |
| **Gene** | **HBV5d+/HBV5d- Fc** | **P-value** |
| STX3 | 2.460390937 | 0.001048905 |
| MMP24OS | 1.734415837 | 0.016867078 |
| GATAD2B | 2.448118237 | 0.003999992 |
| CTSD | 1.515199269 | 0.009424975 |
| EHMT1 | 8.699682324 | 0.020955093 |
| TPP1 | 2.110492508 | 0.033679296 |
| PSAP | 4.032443868 | 0.045436727 |
| UBE2D2 | 2.619003306 | 0.010689833 |
| SKP1 | 2.242776705 | 0.006923086 |
| CAMK2D | 3.569505866 | 0.040297255 |
| RHOT2 | 6.587841283 | 0.037835775 |
| NPC2 | 1.858135797 | 0.028308723 |
| PIK3R2 | 13.23836158 | 0.000593074 |
| UBE2L6 | 2.246337802 | 0.014870379 |
| EEF1E1 | 1.501468287 | 0.030748109 |
| CALU | 2.573299941 | 0.044180513 |
| HSBP1 | 2.529746909 | 0.043087233 |
| TIPRL | 1.835914177 | 0.004056294 |
| UTS2 | 4.936689863 | 0.013389062 |
| TDP2 | 8.790076482 | 0.016040575 |
| BAG2 | 3.579302715 | 0.018175235 |
| HSPB1 | 1.615241954 | 0.005276562 |
| RPLP2 | 2.910113391 | 0.001696658 |
| KRT18 | 1.906651514 | 0.023629168 |
| SERPINE2 | 2.463424342 | 0.00536186 |
| CALM3 | 1.837210039 | 0.034088247 |
| GATD3A | 1.69635617 | 0.037305026 |
| GAA | 1.854827141 | 0.004690072 |
| CTSA | 1.705199124 | 0.013970242 |
| IGF2R | 1.561258553 | 0.034007534 |
| PCNA | 2.300847417 | 0.009147885 |
| CKB | 2.693277402 | 0.027663847 |
| GNS | 1.577937677 | 0.032601594 |
| HSPA6 | 2.278808431 | 0.005962384 |
| PGAM1 | 2.181065883 | 0.010230129 |
| POLR2C | 1.864505532 | 0.01734674 |
| MPST | 1.769948721 | 0.00368781 |
| YWHAQ | 3.957706172 | 0.01143178 |
| GRN | 5.785976241 | 0.011411092 |
| PRDX5 | 2.369540326 | 0.019580588 |
| PRDX3 | 1.583118372 | 0.003453936 |
| CMPK1 | 4.767459119 | 0.030188494 |
| NUP62 | 2.061614249 | 0.001970444 |
| CETN2 | 1.925662738 | 0.043034456 |
| RANBP1 | 1.997156376 | 0.046939624 |
| TKTL1 | 3.941667167 | 0.004930541 |
| CAPZA1 | 2.070077269 | 0.023863528 |
| CTSC | 2.566897859 | 0.032063668 |
| VCP | 3.093752469 | 0.019571981 |
| HNRNPK | 1.641080071 | 0.046697895 |
| YWHAE | 3.513605531 | 0.019117252 |
| PPP2CB | 1.953257027 | 0.038019547 |
| PPIA | 2.728298487 | 0.015150227 |
| YWHAZ | 2.579221765 | 0.047706951 |
| DYNLT1 | 4.866509264 | 0.023973834 |
| SF3A3 | 2.364676181 | 0.006909366 |
| CBX3 | 1.81478229 | 0.04527545 |
| CTBP1 | 1.6112319 | 0.040048637 |
| GAMT | 1.647406886 | 0.036180495 |
| GOLGB1 | 1.575252891 | 0.048255481 |
| NUMA1 | 1.513135719 | 0.038375699 |
| PDIA6 | 1.591269534 | 0.038293958 |
| PLEC | 1.53275881 | 0.004847512 |
| NDUFA5 | 3.154713092 | 0.047210012 |
| FAM98B | 1.676878988 | 0.027134673 |
| FKBP15 | 1.843587386 | 0.011255325 |
| AK1 | 2.006310155 | 0.043148584 |
| HUWE1 | 2.602330579 | 0.009484608 |
| PAF1 | 1.922181435 | 0.038149961 |
| SMARCC2 | 2.25987946 | 0.024949174 |
| NUP210 | 2.478039257 | 0.021324506 |
| DHX38 | 2.085926807 | 0.035787754 |
| STAM | 1.549534731 | 0.007349944 |
| TRIOBP | 2.612381453 | 0.026910138 |
| CHMP4B | 8.190547281 | 0.013060402 |
| ATXN10 | 1.573235753 | 0.013050818 |
| SAE1 | 2.202964189 | 0.036919759 |
| CTSZ | 2.596758807 | 0.029703909 |
| BAZ1B | 1.639976809 | 0.005917859 |
| NUDT5 | 1.794291253 | 0.027819986 |
| MTUS1 | 2.291119911 | 0.00757068 |
| TMCO1 | 2.929961009 | 0.011519227 |
| SNX7 | 1.721062237 | 0.049470458 |
| RTRAF | 2.247306827 | 0.049471486 |
| NUBP2 | 3.160530224 | 0.04888569 |
| PSPC1 | 2.559950687 | 0.032518081 |
| OSBPL9 | 3.69996889 | 0.04482581 |
| BBS4 | 379.2146869 | 0.004063712 |
| STX4 | 5.274595817 | 0.006620904 |
| WDR37 | 17.8118153 | 0.024381334 |
| TOM1L1 | 41.8298221 | 0.000129434 |
| TBC1D4 | 6.214235724 | 0.030537253 |
| FLNB | 11.74112633 | 0.018317607 |
| TTC4 | 14.61384223 | 0.016726161 |
| SLC2A1 | 2.407557775 | 0.037257406 |
| INTS1 | 1.716825557 | 0.030981741 |
| CDKN2AIPNL | 8.288970334 | 0.012179073 |
| DCAF5 | 22.31256579 | 0.017896408 |
| PPA2 | 1.74877019 | 0.003130298 |
| SLC35D1 | 1.674885767 | 0.012456424 |
| DELEC1 | 22.96380234 | 0.003182603 |
| CEP131 | 3.238278413 | 0.030782224 |
| LRRFIP2 | 1.983714622 | 0.047547939 |

| **Table S3 Anti-SMARCC2 antibody enriched genes** | | | |
| --- | --- | --- | --- |
| width | annotation | Gene name | Distance  to TSS |
| 300 | Promoter (<=1kb) | AL732372.3 | -484 |
| 629 | Promoter (<=1kb) | ISG15 | 0 |
| 366 | Promoter (2-3kb) | SCNN1D | 2110 |
| 234 | Intron (ENST00000401095/ENSG00000215912, intron 6 of 8) | MMEL1 | -19226 |
| 200 | Intron (ENST00000401095/ENSG00000215912, intron 6 of 8) | MMEL1 | -36406 |
| 197 | Intron (ENST00000401095/ENSG00000215912, intron 6 of 8) | AC242022.1 | -14794 |
| 200 | Promoter (<=1kb) | CLSTN1 | 129 |
| 206 | Promoter (<=1kb) | LINC01778 | 0 |
| 240 | Intron (ENST00000634606/ENSG00000172456, intron 6 of 16) | FGGY | 5074 |
| 274 | Distal Intergenic | AC242852.1 | -140373 |
| 563 | Distal Intergenic | AC242852.1 | -128318 |
| 204 | Distal Intergenic | AC242852.1 | -123262 |
| 194 | Distal Intergenic | AC242852.1 | -112744 |
| 200 | Distal Intergenic | AC242852.1 | -103555 |
| 422 | Distal Intergenic | AC242852.1 | -91130 |
| 217 | Distal Intergenic | AC242852.1 | -90011 |
| 555 | Distal Intergenic | AC242852.1 | -88204 |
| 197 | Promoter (<=1kb) | RNA5S6 | -835 |
| 197 | Promoter (<=1kb) | RNA5S10 | -958 |
| 216 | Promoter (1-2kb) | PFKP | 1321 |
| 220 | Promoter (<=1kb) | MIR4480 | -416 |
| 236 | Promoter (<=1kb) | ZFYVE27 | 0 |
| 211 | Promoter (<=1kb) | PSTK | 0 |
| 443 | Promoter (<=1kb) | PPP2R2D | 0 |
| 216 | Promoter (<=1kb) | DUX4L25 | -480 |
| 265 | Promoter (<=1kb) | AC131934.1 | -907 |
| 791 | Promoter (<=1kb) | KRTAP5-AS1 | 0 |
| 311 | Promoter (<=1kb) | TEAD1 | -170 |
| 223 | Distal Intergenic | OR4C50P | -399872 |
| 322 | Promoter (<=1kb) | NEAT1 | -382 |
| 255 | Promoter (<=1kb) | YIF1A | 0 |
| 351 | Promoter (<=1kb) | TMEM151A | 154 |
| 373 | Promoter (<=1kb) | PC | 785 |
| 376 | Promoter (<=1kb) | KMT5B | 0 |
| 255 | Promoter (<=1kb) | PAK1 | 48 |
| 197 | Promoter (<=1kb) | MAGOHB | 0 |
| 210 | Distal Intergenic | AK6P1 | 642687 |
| 378 | Promoter (2-3kb) | MYL6 | 2585 |
| 337 | 3' UTR | MYL6 | 3562 |
| 1616 | Promoter (<=1kb) | SMARCC2 | 0 |
| 260 | Distal Intergenic | LINC00485 | 92994 |
| 218 | Promoter (<=1kb) | VPS37B | -7 |
| 279 | Promoter (1-2kb) | AL133304.1 | 1366 |
| 223 | Promoter (<=1kb) | LINC02317 | 419 |
| 264 | Promoter (<=1kb) | LINC02321 | 0 |
| 670 | Promoter (<=1kb) | KLC1 | 0 |
| 359 | Intron (ENST00000553757/ENSG00000258913, intron 1 of 2) | LINC02691 | 27874 |
| 210 | Distal Intergenic | FP325313.1 | -210544 |
| 201 | Distal Intergenic | PPP1R1AP2 | 2364144 |
| 205 | Promoter (<=1kb) | ENKD1 | 187 |
| 228 | Promoter (<=1kb) | IFT20 | 0 |
| 214 | Promoter (1-2kb) | U2 | 1297 |
| 217 | Promoter (2-3kb) | RN7SL258P | -2730 |
| 279 | Intron (ENST00000577161/ENSG00000141564, intron 13 of 21) | RPTOR | -46648 |
| 214 | Distal Intergenic | - | -295869 |
| 610 | Promoter (<=1kb) | ATP9B | -122 |
| 323 | Promoter (2-3kb) | WDR18 | 2402 |
| 267 | Promoter (1-2kb) | ATP5F1D | -1365 |
| 211 | Intron (ENST00000262965/ENSG00000071564, intron 5 of 18) | TCF3 | -6189 |
| 330 | Intron (ENST00000617428/ENSG00000268861, intron 7 of 21) | ARHGEF18 | 3668 |
| 444 | Promoter (<=1kb) | RAB11B | 0 |
| 205 | Promoter (<=1kb) | MYO1F | 212 |
| 388 | Promoter (<=1kb) | RFX1 | 0 |
| 233 | Promoter (<=1kb) | JUND | -16 |
| 212 | Distal Intergenic | AC112702.1 | 813033 |
| 205 | Distal Intergenic | ZNF565 | -23071 |
| 397 | Distal Intergenic | ZNF565 | -25304 |
| 265 | Distal Intergenic | AC012617.1 | -14250 |
| 193 | Distal Intergenic | ZNF875 | -19194 |
| 208 | Intron (ENST00000440698/ENSG00000231918, intron 6 of 10) | CRYGGP | -5101 |
| 376 | Promoter (<=1kb) | DOK1 | 66 |
| 310 | Distal Intergenic | LINC01943 | 29840 |
| 350 | Distal Intergenic | LINC01943 | 26645 |
| 394 | Distal Intergenic | LINC01943 | 21902 |
| 223 | Distal Intergenic | LINC01943 | 20696 |
| 231 | Distal Intergenic | AC233266.1 | -79632 |
| 288 | Distal Intergenic | AC233266.1 | -54975 |
| 209 | Distal Intergenic | LINC02631 | 17342 |
| 206 | Distal Intergenic | RNU6-187P | 4832 |
| 219 | Promoter (<=1kb) | ALS2 | -39 |
| 351 | Distal Intergenic | AC079612.2 | 18869 |
| 264 | Distal Intergenic | MIR663AHG | -793735 |
| 326 | Distal Intergenic | FRG1CP | 106857 |
| 228 | Promoter (<=1kb) | ZNF335 | -45 |
| 308 | Intron (ENST00000606208/ENSG00000272259, intron 1 of 1) | LINC00029 | 7054 |
| 237 | Promoter (<=1kb) | MIR6724-1 | -48 |
| 207 | Promoter (<=1kb) | MIR3687-1 | 0 |
| 210 | Promoter (<=1kb) | FP671120.3 | 61 |
| 500 | Promoter (2-3kb) | RNA5-8SN2 | 2702 |
| 196 | Intron (ENST00000623664/ENSG00000278996, intron 2 of 6) | RNA5-8SN2 | 7062 |
| 252 | Intron (ENST00000623664/ENSG00000278996, intron 2 of 6) | RNA5-8SN2 | 7793 |
| 241 | Exon (ENST00000623664/ENSG00000278996, exon 3 of 7) | RNA5-8SN2 | 10448 |
| 211 | Intron (ENST00000623664/ENSG00000278996, intron 5 of 6) | RNA5-8SN2 | 13213 |
| 509 | Promoter (1-2kb) | RNA5-8S5 | 1143 |
| 562 | Promoter (2-3kb) | RNA5-8S5 | 2685 |
| 203 | Distal Intergenic | RNA5-8S5 | 3720 |
| 196 | Promoter (<=1kb) | FP236383.2 | 0 |
| 359 | Promoter (<=1kb) | RNA5-8SN3 | 211 |
| 196 | Intron (ENST00000623860/ENSG00000280441, intron 2 of 6) | RNA5-8SN3 | 7025 |
| 410 | Distal Intergenic | RNA5-8SN3 | 17803 |
| 648 | Distal Intergenic | MIR6724-4 | -14149 |
| 277 | Promoter (<=1kb) | pRNA | 46 |
| 210 | Promoter (<=1kb) | FP236383.3 | 59 |
| 328 | Promoter (2-3kb) | RNA5-8SN1 | 2506 |
| 436 | Distal Intergenic | RNA5-8SN1 | 3721 |
| 231 | Distal Intergenic | RNA5-8SN1 | 10401 |
| 214 | Distal Intergenic | RNA5-8SN1 | 27996 |
| 223 | Promoter (<=1kb) | MIR3687-2 | 0 |
| 197 | Distal Intergenic | AP001464.1 | 846712 |
| 234 | Distal Intergenic | AL022324.2 | 4554 |
| 193 | Intron (ENST00000358763/ENSG00000185666, intron 4 of 13) | SYN3 | 19329 |
| 264 | Promoter (<=1kb) | JOSD1 | 0 |
| 245 | Intron (ENST00000446281/ENSG00000227110, intron 3 of 5) | AC023481.1 | -45577 |
| 241 | Intron (ENST00000446281/ENSG00000227110, intron 3 of 5) | AC023481.1 | -46089 |
| 255 | Promoter (<=1kb) | LINC02585 | 224 |
| 209 | Promoter (<=1kb) | SEMA3B | 565 |
| 243 | Distal Intergenic | RNU6-488P | -1161005 |
| 252 | Promoter (<=1kb) | CMSS1 | 0 |
| 196 | Promoter (<=1kb) | INAVAP1 | 0 |
| 212 | Promoter (2-3kb) | FAM53A | -2244 |
| 222 | Promoter (<=1kb) | ACOX3 | 0 |
| 364 | Distal Intergenic | AC118282.3 | 53969 |
| 421 | Distal Intergenic | SNX18P25 | 122579 |
| 196 | Distal Intergenic | SNX18P25 | 671492 |
| 226 | Promoter (<=1kb) | CCNI | -91 |
| 270 | Promoter (<=1kb) | DUX4L4 | 0 |
| 246 | Promoter (<=1kb) | DUX4L1 | -487 |
| 201 | Promoter (<=1kb) | DUX4L2 | -529 |
| 285 | Promoter (2-3kb) | DUX4 | 2989 |
| 196 | Promoter (2-3kb) | DUX4 | -2421 |
| 219 | Distal Intergenic | EMB | 1002836 |
| 210 | Distal Intergenic | EMB | 798379 |
| 233 | Distal Intergenic | EMB | 785344 |
| 363 | Distal Intergenic | EMB | 750197 |
| 203 | Intron (ENST00000523189/ENSG00000204764, intron 14 of 27) | RANBP17 | 54601 |
| 323 | Promoter (<=1kb) | CLTB | 0 |
| 234 | Promoter (<=1kb) | RNF44 | 0 |
| 463 | 5' UTR | DBN1 | 5629 |
| 407 | Intron (ENST00000390654/ENSG00000050767, intron 2 of 28) | RN7SL646P | 17458 |
| 277 | Promoter (1-2kb) | FLT4 | -1996 |
| 278 | Promoter (<=1kb) | FOXQ1 | 281 |
| 257 | Intron (ENST00000357250/ENSG00000112280, intron 4 of 37) | COL9A1 | 7389 |
| 365 | Intron (ENST00000400788/ENSG00000215712, intron 3 of 3) | LDHAL6FP | -10471 |
| 349 | Distal Intergenic | AL513210.1 | 19731 |
| 346 | Intron (ENST00000630384/ENSG00000112584, intron 6 of 10) | FAM120B | -26815 |
| 281 | Promoter (2-3kb) | ADAP1 | -2180 |
| 503 | Intron (ENST00000404826/ENSG00000146555, intron 13 of 44) | SDK1 | 5378 |
| 293 | Distal Intergenic | AC093392.2 | -7221 |
| 195 | Distal Intergenic | - | -1024315 |
| 239 | Distal Intergenic | - | -1675548 |
| 273 | Distal Intergenic | AC128676.1 | 1963663 |
| 250 | Distal Intergenic | CICP24 | 18464 |
| 595 | Promoter (<=1kb) | TRIP6 | 0 |
| 196 | Intron (ENST00000284629/ENSG00000154438, intron 4 of 12) | ASZ1 | 8495 |
| 368 | Promoter (<=1kb) | UBE2H | 0 |
| 269 | Promoter (<=1kb) | NUDT18 | -105 |
| 262 | Promoter (1-2kb) | REXO1L2P | 1903 |
| 229 | Distal Intergenic | - | -730444 |
| 282 | Distal Intergenic | - | -1737665 |
| 228 | Distal Intergenic | - | -1832468 |
| 230 | Promoter (<=1kb) | SPATA31C1 | 350 |
| 254 | Promoter (<=1kb) | TRMO | 0 |
| 220 | Promoter (<=1kb) | CEL | 886 |
| 275 | Distal Intergenic | LINC01451 | 17020 |
| 1079 | Intron (ENST00000462942/ENSG00000181090, intron 20 of 21) | MIR602 | 6811 |
| 195 | Promoter (<=1kb) | USP9X | -832 |
| 448 | Distal Intergenic | DUX4L17 | -3734 |
| 316 | Promoter (2-3kb) | DUX4L18 | -2514 |
| 480 | Promoter (1-2kb) | DUX4L19 | -1934 |
| 287 | Promoter (<=1kb) | AL162713.1 | -52 |
| 338 | Intron (ENST00000260403/ENSG00000137842, intron 11 of 13) | EPB42 | 3690 |
| 269 | Promoter (<=1kb) | MT-TF | -229 |
| 850 | Promoter (<=1kb) | MT-RNR1 | 0 |
| 349 | Promoter (<=1kb) | MT-RNR2 | 394 |
| 216 | Promoter (<=1kb) | MT-CO1 | 0 |
| 200 | Promoter (<=1kb) | MT-TG | -153 |
| 655 | Promoter (<=1kb) | MT-ND5 | 493 |
| 225 | Promoter (<=1kb) | MT-CYB | 201 |
